# Irisin maintains ER homeostasis and activates AMPK via integrin αVβ5 to attenuate CSE + LPS-induced emphysema and inflammation

**DOI:** 10.1186/s12931-026-03850-9

**Published:** 2026-07-30

**Authors:** Zina Bai, Tongxinwei Sun, Zelin Chen, Siqin Han, Jingwen Li, Xiaopeng Zhang, Cuiqing Ma, Yahong Chen, Ping Jiang, Xixin Yan, Aihong Meng

**Affiliations:** 1 The Third Department of Pulmonary and Critical Care Medicine, The Second Hospital of Hebei Medical University, Shijiazhuang, Hebei China; 2 The First Department of Pulmonary and Critical Care Medicine, The Second Hospital of Hebei Medical University, Shijiazhuang, Hebei China; 3 Hebei Key Laboratory of Respiratory Critical Care Medicine, Shijiazhuang, Hebei China; 4 Department of Thoracic Surgery, Hebei General Hospital, Shijiazhuang, Hebei China; 5 Department of Immunology, Key Laboratory of Immune Mechanism and Intervention On Serious Disease in Hebei Province, Hebei Medical University, Shijiazhuang, Hebei China; 6 Department of Pulmonary and Critical Care Medicine, Peking University Third Hospital, Beijing, China; 7 Department of Pulmonary and Critical Care Medicine, Tianjin First Central Hospital, Tianjin, China

**Keywords:** Irisin, Integrin αVβ5, ER stress, AMPK, Emphysema, Inflammation

## Abstract

**Background:**

AECOPD adversely affects patient survival rates and overall quality of life. Irisin is being increasingly recognized for its therapeutic potential in attenuating pulmonary injury, but its underlying mechanism remains unclear.

**Methods:**

Human lung tissue samples were subjected to IHC analysis for irisin, integrin αVβ5, and GRP78 expression. CSE+LPS-induced mouse and cell models were used to investigate whether irisin protects against emphysema and inflammation by regulating ER homeostasis and AMPK activity via integrin αVβ5.

**Results:**

In COPD patients, irisin levels are decreased, whereas integrin αVβ5 and GRP78 levels are elevated. Irisin improved lung function and attenuated emphysema and inflammation in mice, and these effects were abolished by cilengitide. Irisin directly bound to integrin αVβ5, maintained ER homeostasis via the PERK/ATF4/CHOP pathway, and activated AMPK. These protective effects were lost upon integrin αVβ5 knockdown.

**Conclusion:**

These findings suggest that through integrin αVβ5, irisin concurrently maintains ER homeostasis and activates AMPK, thereby alleviating CSE+LPS-induced emphysema and inflammation, suggesting a novel therapeutic direction for the management of AECOPD.

**Supplementary Information:**

The online version contains supplementary material available at https://doi.org/10.1186/s12931-026-03850-9.

## Introduction

Chronic obstructive pulmonary disease (COPD) is a progressive and irreversible respiratory condition disorder characterized by persistent airflow limitation. Representing a major global public health challenge, COPD consistently ranks among the leading chronic diseases worldwide in terms of both prevalence and mortality, with 90% of deaths occurring in developing countries [1–3]. Cigarette smoke (CS) is widely recognized as the primary aetiological factor for COPD, while bacterial infections significantly predispose individuals to acute exacerbations of COPD (AECOPD). Clinical evidence indicates that COPD patients who experience frequent acute exacerbations face a mortality risk that is four to five times higher than that of patients without exacerbations, thereby severely compromising survival and quality of life [4, 5]. Consequently, the development of effective prevention and treatment strategies for AECOPD is of paramount clinical significance and social value, as it aims to reduce patient mortality and improve overall health outcomes.

Irisin was first discovered and reported by Harvard Medical School in 2012 [6]. It is a myokine encoded by the FNDC5 gene, which is a target of PGC-1α (peroxisome proliferator-activated receptor-γ coactivator-1α). After cleavage, it is secreted as a protein [7]. Irisin produced by skeletal muscle during exercise enters lung tissue through blood circulation and exerts a protective effect [8]. Studies have demonstrated that serum irisin has the potential to alleviate emphysema symptoms in COPD patients and to regulate epithelial apoptosis [9]; it also has protective effects against damage via the Nrf2/HO-1 signalling pathway [10].

Our previous investigations revealed that irisin ameliorates PM2.5-induced acute lung injury through modulation of the Nod2/NF-κB pathway, and AMPK/mTOR signalling pathway and through inhibition of alveolar macrophage autophagy and pyroptosis [11–13]. Our preliminary findings also indicate that irisin can mitigate lung injury caused by cigarette smoke extract (CSE) combined with lipopolysaccharide (LPS) through activation of the AMPK-Beclin1 autophagy pathway [14]. Studies have identified the integrin αVβ5 complex as a key functional receptor for irisin in the context of bone-forming cells and gut epithelial cells [7, 15, 16]. Integrins constitute a major class of important transmembrane proteins that, upon activation, can participate in apoptosis, gene expression, and cell differentiation via multiple signalling pathways [17]. However, previous studies on irisin have largely focused on its anti-inflammatory effects. Whether its regulatory effects on AECOPD are mediated through integrin αVβ5 and subsequently involve the AMPK pathway and other signalling cascades remain unexplored.

Endoplasmic reticulum (ER) homeostasis refers to the dynamic equilibrium of protein folding, calcium ion concentration, and redox states within the ER [18]. When this balance is disrupted, cells initiate the unfolded protein response (UPR) to restore homeostasis; however, persistent imbalance can trigger endoplasmic reticulum stress (ERS) [19, 20]. The ER is the largest calcium reservoir in cells and is responsible for regulating the balance of calcium ions within cells. The ER lumen maintains a relatively high Ca²⁺ concentration, which is essential for the activity of molecular chaperones such as GRP78/BiP. When the ER is subjected to stress, calcium ions are forced to leak into the cytoplasm, resulting in calcium overload, which triggers a series of destructive reactions [21, 22]. Pathogenic stimuli such as CSE and LPS can activate the UPR, leading to a series of pathological changes, including chronic airway inflammation, epithelial apoptosis, hypersecretion of mucus, and structural damage to the alveoli [23–25]. Upon pathogenic stimulation, significant Ca²⁺ release from the ER lumen results in the abnormal accumulation of misfolded proteins and the upregulation of GRP78 expression, ultimately leading to mucus and alveolar structural damage [26]. According to previous reports, CS can activate the ER-related signalling pathway PERK/ATF4/CHOP, leading to airway remodelling and the development of COPD [27, 28]. The stability of ER function plays a crucial role in the progression of COPD [29, 30]. Currently, whether irisin, by binding to integrin αVβ5, modulates ER homeostasis and AMPK activity remains unclear. This unresolved mechanism represents a critical research gap that urgently needs to be addressed in this field.

This study aimed to verify that irisin can maintain ER homeostasis and activate AMPK by binding to integrin αVβ5, thereby alleviating CSE + LPS-induced emphysema and airway inflammation. These findings clarify a new irisin regulatory mechanism in AECOPD. The results of this study are anticipated to identify an innovative prospective target for the treatment of AECOPD, developing a theoretical framework to inform the creation of innovative therapeutic approaches for this advancing respiratory condition.

## Materials and methods

### Materials and reagents

Integrin αV (1:1000, CY5317), ATF4 (1:1000, CY5873), and CHOP (1:1000, CY6694) were purchased from Abways (Beijing, China); p-PERK (1:5000, 82534-1-RR), GRP78 (1:5000, 11587-1-AP), and Irisin (1:2000, 23995-1-AP) were obtained from Proteintech (Wuhan, China); Camkk2 (1:1000, HY-P81205) was obtained from MedChemExpress (New Jersey, USA); p-Camkk2 (1:1000, AF4487) was obtained from Affinity (Jiangsu, China); Integrin β5 (1:1000, 3629T), AMPK (1:1000, 5831T), and p-AMPK (1:1000, 2535T) were obtained from CST (Danvers, MA, USA); Irisin (1:200, bs-8486R) was obtained from Signalway Antibody (Nanjing, China); PERK (1:1000, F0027) was purchased from Sellect (Houston, USA); Integrin αVβ5 (1:200, sc-81632) was obtained from Santa Cruz (Texas, USA), and another Integrin αVβ5 antibody (1:200, bs-1356R) was purchased from Bioss (Beijing, China). Cilengitide TFA (HY-16143), Thapsigargin (HY-13433), and BAPTA-AM (HY-100545) were purchased from MedChemExpress (New Jersey, USA). Diamond filter cigarettes (11 mg tar, 1 mg nicotine, 13 mg carbon monoxide) were provided by Tobacco Industry Company (Hebei, China). LPS(L2630) was purchased from Sigma (MO, USA). Recombinant irisin protein (11451) was obtained from Cayman Chemical Company (Michigan, USA).

### Patients and lung tissue

A total of 25 patients were divided into three groups: 8 non-smokers, 9 smokers with normal lung function, and 8 smokers with COPD, according to the Global Initiative for Chronic Obstructive Lung Disease (GOLD) guideline [31]. To avoid tumor contamination, lung tissue specimens were harvested from areas at least 5 cm away from the tumor margin at Hebei General Hospital. Histopathological examination of biopsy specimens was performed on paraffin-embedded sections to confirm the pathological features of each lung tissue sample. This study was approved by the Medical Science Research Ethics Committee of Hebei General Hospital (Approval No.: 2023 − 321), and all participants signed a written informed consent form before participation. Detailed clinical characteristics of patients are presented in Table 1.

**Table 1:** Clinical characteristics of three groups

	Nonsmokers	Smokers	COPD	*P* value
Sex (F/M)	3/5	7/2	5/3	0.115
Age, y	58.63 ± 16.18	60.00 ± 8.185	66.75 ± 6.671	0.304
BMI	26.28 ± 4.216	25.76 ± 2.744	24.46 ± 2.816	0.535
FEV1/FVC, %	80.88 ± 77.36	77.78 ± 44.10	62.38 ± 26.15	< 0.0001
FEV1 pred, %	109.0 ± 14.63	104.2 ± 18.25	71.97 ± 13.12	0.0002
Comorbidity(Y/N)	2/6	4/5	4/4	0.561

Values are presented as the Mean ± SD

### Immunohistochemistry (IHC)

Human lung tissue samples were embedded in paraffin and sectioned into slices with a thickness of 4 μm. These lung tissues were incubated with primary antibodies targeting irisin (23995-1-AP, Proteintech), Integrin αVβ5 (bs-1356R, Bioss), and GRP78 (11587-1-AP, Proteintech). Following this, the sections were incubated with secondary antibody, then subjected to staining with DAB and counterstaining with hematoxylin to visualize cell nuclei. The stained sections were dehydrated through a graded ethanol series and subsequently mounted using neutral balsam. Microscopic images were acquired at 200×magnification utilizing a Zeiss light microscope (Germany). Quantitative analysis of the expression levels of the target proteins was conducted using ImageJ software. For each section, a minimum of five representative visual fields of view were selected for analysis, and the Integrated Density (IntDen) values were quantified and used for statistical analysis.

### Data collection and analysis of ERS-Related DEGs

Data were obtained from the Gene Expression Omnibus (GEO) database hosted by the National Center for Biotechnology Information (NCBI) (https://www.ncbi.nlm.nih.gov/geo/). The GSE38974 dataset (GPL4133 platform), includes 23 COPD lung tissue samples and 9 normal lung tissue samples. The GSE69818 dataset (also GPL4133) includes 32 lung tissue samples from COPD patients with emphysema. Differential expression analysis was conducted using a false discovery rate (FDR) threshold of less than 0.05. Data reproducibility was assessed via principal component analysis (PCA). A total of 785 genes associated with ERS, each exhibiting a relevance score of 7 or above, were retrieved from the GeneCards database to construct prognostic gene signatures. Differentially expressed genes (DEGs) related to ERS were identified utilizing the “limma” package within the R software environment. The selection criteria included an adjusted p-value of less than 0.05 and an absolute fold change exceeding 1.5. Visualization, including volcano plots, heatmaps, box plots, and receiver operating characteristic (ROC) curves, was performed using the “heatmap,” “ggplot2,” and “pROC” packages within the R environment.

### Animals and treatments

Male C57BL/6J mice, aged 4–6 weeks, weighing between 20 and 25 g, were obtained from the Experimental Animal Center of Hebei Medical University. The animals were maintained under specific pathogen-free (SPF) conditions, with free access to food and water, and were maintained under a 12-hour light/dark cycle. A total of forty male C57BL/6J mice were randomly divided into 5 groups (*n* = 8 per group): (1) Control group; (2) CSE + LPS group; (3) CSE + LPS+Irisin group; (4) CSE + LPS+Irisin+Cilengitide group; (5) CSE + LPS+Cilengitide group.

The mouse model of AECOPD was established by combined induction with CSE and LPS, referring to previously published protocols [14, 32–34]. Mice in the control group were maintained under identical environmental conditions without exposure to CSE and were administered intratracheal instillation (i.t.) of 20 µL phosphate-buffered saline (PBS) on Days 1, 14, and 28. Starting at Week 3, irisin (0.5 µg/g, 100 µL) [13, 14] was administered via intraperitoneal (i.p.) injection three times per week, 30 min prior to CSE exposure or LPS (1 µg/g, 20 µL)instillation. The integrin αVβ5 inhibitor cilengitide (5 µg/g, 100 µL) was administered via i.p. injection three times per week [35], 30 min prior to irisin injection. Mice in the groups not receiving irisin and/or cilengitide were injected i.p. with 100 µL PBS at corresponding time points to serve as vehicle controls.

The body weight of each mouse was measured and documented on a weekly basis throughout the duration of the experimental period. On day 29, 3 mice from each group were randomly selected for photoacoustic imaging and lung function testing. All mice were euthanized, and lung tissues, serum, and bronchoalveolar lavage fluid (BALF) samples were immediately harvested for subsequent analyses. All experimental protocols involving animals were approved by the Animal Care and Use Committee of the Second Hospital of Hebei Medical University (Approval No.: 2024-AE289) and complied with the National Institutes of Health (NIH) Guidelines for the Care and Use of Laboratory Animals.

### Lung function measurement

Pulmonary function was assessed utilizing a small animal spirometer (FlexiVent system, USA) following a previously established protocol with slight modifications [36]. Briefly, all mice were weighed and subsequently anesthetized via i.p. administration of Avertin at a dosage of 10 µL/g. Following anesthesia, a tracheostomy was performed, and the trachea was cannulated. The instrument was then connected to assess pulmonary function. Measurements obtained included forced expiratory volume at 0.1 s (FEV0.1), forced vital capacity (FVC), and peak expiratory flow (PEF).

### Lung pathological analysis

Human and murine lung tissues were embedded in paraffin and sectioned into 4 μm thick slices for hematoxylin and eosin (HE) staining. Emphysematous lesions were assessed by measuring the mean linear intercept (MLI) and the destruction index (DI). For each mouse, analysis was conducted on 10 randomly selected fields per slide using a light microscope. Quantification of these indices was performed either manually or with the assistance of Image Pro Plus 6.0 software. For each parameter, both average values were calculated and the resulting mean values were subsequently analyzed using statistical methods [37].

### Photoacoustic imaging (PAI)

The thigh and tail areas of the mice were shaved before the experiment to remove hair from the imaging region. After anesthesia, the mice were fixed on the device using medical tape and placed into the imaging chamber for photoacoustic imaging of the thigh and tail regions. The selected wavelength for photoacoustic imaging was 700–850 nm. The photoacoustic imaging and oxygen saturation levels of the mice’s skeletal muscle area and tail area were measured using the Marsonics PIIP-FC (Tsingpai Technology Co,.LTD).

### Preparation of CSE

The CSE solution was formulated following established protocols outlined in previous studies [14, 38]. Specifically, two cigarettes were positioned on the duct and connected to a single-channel intelligent smoking machine. The smoke generated was captured into 10 mL of RPMI 1640 medium and subjected to repeated aspiration to ensure complete dissolution, resulting in a homogeneous suspension. The CSE was subsequently sterilized by filtration through a 0.22 μm membrane filter and adjusted to a pH range of 7.2 to 7.4. The concentration of the solution was standardized using an enzymatic spectrophotometric assay at 320 nm, with this measurement defined as 100%.

### Cell culture and treatments

Mouse alveolar macrophages (MH-S cell line) utilized in this study were obtained from the American Type Culture Collection (ATCC, USA). The cells were cultured in RPMI 1640 medium supplemented with 10% fetal bovine serum (FBS; Gibco, USA) and subsequently incubated at 37 °C in an atmosphere containing 5% CO2 and 95% air until reaching the appropriate confluency for experimental procedures. The drug concentrationa were consistent with that determined by the research group in previous studies [14]. For treatment, cells were exposed to LPS at 10 µg/mL in combination with 2% CSE for a duration of 12 h. Before LPS and CSE exposure, MH-S cells were pretreated for 30 min with irisin (20 nM), Thapsigargin (1 µM) [39, 40], or BAPTA-AM (10 µM) [41, 42].

### Integrin αV and Integrin β5 siRNA transfection

The siRNA transfection procedure was conducted in accordance with the method previously described [14]. Specifically, integrin αV-targeting small interfering RNAs (siRNAs) with the sequences 5′-GACCCGUUGUCACUGUAAATT-3′ and 5′-UUUACAGUGACAACGGGUCTT-3′, integrin β5-targeting siRNAs with the sequences 5′-CUGCCAAGAUGGCAUAUCUTT-3′ and 5′-AGAUAUGCCAUCUUGGCAGTT-3′, as well as nonspecific siRNA controls (GenePharma), were employed to transfect MH-S cells.

### Surface plasmon resonance (SPR)

The MH-S cells were divided into the siNC group and the siαVβ5 group. The treated MH-S cells were added with an appropriate amount of pre-cooled RIPA, centrifuged, and the supernatant was extracted. The supernatants were adjusted to the same concentration for SPR experiments. The recombinant irisin protein was diluted and immobilized on the chip surface. The supernatants of the siNC group and siαVβ5 group cells, which were adjusted to the same concentration, were successively placed in the sample rack of the SPR biosensor. The Biacore™ 1 K system was used to record the binding curves of irisin and integrin αVβ5 in the cell lysate supernatant in real time (RU value changes) for the detection of the binding affinity of the cell lysate supernatant.

### Coimmunoprecipitation (Co-IP)

Cells were lysed on ice utilizing a lysis buffer. An aliquot of 50 µL of the resulting cell lysate was reserved as the input control, and the remaining lysate was divided into the IgG group and the IP group. These need to be incubated overnight with the anti-integrin αVβ5 antibody (1:200, sc-81632) or the IgG antibody (Beyotime) at 4 °C. Immune complexes were subsequently isolated through Protein A/G magnetic beads (88803, Thermo Scientific) and incubated with gentle agitation for two hours at room temperature. After thorough washing to eliminate non-specific proteins, samples were heated to elute the bound proteins. Post-centrifugation, the supernatant was harvested. The primary antibodies were employed for Western blotting: anti-integrin αV (1:1000, CY5317), anti-integrin β5 (1:1000, 3629T), and anti-irisin (1:2000, 23995-1-AP).

### Immunofluorescence (IF)

Lung tissues (4 μm thickness) and MH-S cells were fixed and then blocked with goat serum for 30 min at 23 °C. The samples were then incubated with the primary antibodies. For cells: anti-irisin (1:200, 23995-1-AP), anti-GRP78 (1:500, 11587-1-AP), anti-Integrin αVβ5 (1:200, sc-81632). For lung tissues: anti-irisin (1:200, 23995-1-AP), anti-GRP78 (1:500, 11587-1-AP), anti-F4/80 (1:200, 28463-1-AP), anti-Integrin αVβ5 (1:200, bs-1356R; Bioss). After primary antibody incubation, the sections were treated with secondary antibodies conjugated with Alexa Fluor 594, Alexa Fluor 488, or Alexa Fluor 647 and counterstained with DAPI. Finally, the stained sections were analyzed using fluorescence microscopy.

### Molecular docking and molecular dynamics simulation

The protein-peptide docking between integrin αVβ5 (PDB ID: 4WK0) and Irisin (PDB ID: 4LSD) was performed using the GRAMM web server (http://gramm.compbio.ku.edu/) to elucidate their interaction dynamics [43]. After preprocessing of the protein structures, the protein surface was discretized into a three-dimensional grid, and rigid-body docking simulations were conducted. From the resulting docking poses, the top ten conformations exhibiting the highest scores were selected for further analysis. Visualization of the three-dimensional structures was accomplished using PyMOL version 2.6 software [44].

Subsequently, the protein complex predicted by AlphaFold modeling was subjected to a 100-ns molecular dynamics (MD) simulation using GROMACS version 2022.03 [45], employing the AMBER99SB-ILDN force field [46]. Analysis of the MD trajectory included calculation of root mean square deviation (RMSD), root mean square fluctuation (RMSF), radius of gyration (Rg), solvent-accessible surface area (SASA), and hydrogen bond (H-bond) metrics to assess the stability and conformational dynamics of the complex. Additionally, Gibbs free energy estimations were derived from RMSD and Rg data using GROMACS tools. To quantify the binding free energy of the protein-ligand complex, molecular mechanics/Poisson-Boltzmann surface area (MM/PBSA) calculations were conducted employing the “MMPBSA.py v.16.0” script [47]. The binding free energy was determined based on the stable segment of the trajectory, specifically the final 20 ns characterized by consistent RMSD values, as evaluated through the MM/PBSA approach.

### Transmission electron microscopy (TEM)

MH-S cells were exposed to various stimulatory agents and subsequently harvested by centrifugation. The cell pellets were fixed in 2.5% glutaraldehyde and then dehydrated through a graded ethanol series after which they were embedded, sectioned, and stained. Ultimately, imaging was conducted utilizing a Hitachi 7500 TEM functioning at an acceleration voltage of 80 kV.

### Determination of Ca^2+^ concentration

To measure intracellular calcium levels, treated MH-S cells were incubated with a solution containing 2 µM Fluo-4 AM (S1060, Beyotime) for 40 min. Thereafter, the samples were thoroughly washed. Following this, an additional incubation period of 20 min was conducted to facilitate the thorough intracellular hydrolysis of Fluo-4 AM to Fluo-4. Thereafter, these cells were incubated with DAPI for 10 min, followed by another washing step. Finally, the cells were analyzed using fluorescence microscopy.

### Western blotting

As previously detailed [14], total protein extracted from lung tissues and MH-S cells was subjected to separation via SDS–PAGE. The primary antibodies employed encompassed Integrin αV (1:1000, CY5317), Integrin β5 (1:1000, 3629T), Irisin (1:2000, bs-8486R), Camkk2 (1:1000, HY-P81205), p-Camkk2(1:1000, AF4487), AMPK(1:1000, 5831T), p-AMPK(1:1000, 2535T), GRP78 (1:5000, 11587-1-AP), PERK (1:1000, F0027), p-PERK (1:5000, 82534-1-RR), ATF4(1:1000, CY5873), and CHOP (1:1000, CY6694). Visualization of target proteins was performed using an imaging system, and subsequent quantification was conducted with ImageJ software.

### Real-time quantitative polymerase chain reaction (qRT–PCR)

The primer sequences (5′–3′) corresponding to the target mRNAs are presented (Table 2). Total RNA was isolated utilizing TriQuick Reagent (Solarbio). Complementary DNA (cDNA) synthesis was conducted using PrimeScript RT Master Mix (Vazyme). Quantitative real-time PCR (qRT-PCR) was performed using the aforementioned primers in conjunction with Hieff qPCR SYBR^®^ Green Master Mix (Vazyme). The mRNA levels within the samples were quantified, and relative gene expression changes were assessed employing the comparative Ct (ΔΔCt) methodology.

**Table 2:** List of primer sequences

Gene	Forward primer (5’→3’)	Reverse primer (3’→5’)
TNF-α	ATGTCTCAGCCTCTTCTCATTC	GCTTGTCACTCGAATTTTGAGA
IL-18	AGACCTGGAATCAGACAACTTT	TCAGTCATATCCTCGAACACAG
IL-10	CAAGGCAGTGGAGCAGGTGAAG	CGCTTTGGTGAGTAGACAGAGGTC
Integrin αV	GAAAGTCCCGCCGAGTATGC	ACCAGGAGAAACATCGAGGAC
Integrin β5	GCCCGTTATGAAATGGCCTC	AGGCGAAATCGACAGTGTGT
GAPDH	GGTTGTCTCCTGCGACTTCA	TGGTCCAGGGTTTCTTACTCC

### Enzyme-linked immunosorbent assays (ELISA)

The levels TNF-α (EK282), IL-18 (EK218), IL-10 (EK210), and Irisin (ZC-55012) in serum, BALF, and cell culture supernatants were measured using commercial ELISA kits.Following the protocols provided by the manufacturers, the absorbance of each sample was recorded at a wavelength of 450 nm utilizing a microplate reader.

### Data analysis

Statistical analyses were conducted utilizing GraphPad Prism version 9.5. The data were evaluated employing the Kolmogorov–Smirnov test or the Shapiro–Wilk test. For comparisons between two groups, unpaired t-tests and Chi-square tests were utilized. In contrast, analyses involving multiple groups were conducted using one-way analysis of variance (ANOVA) followed by Dunnett’s multiple comparison test was performed. A p-value of less than 0.05 was regarded as indicative of statistical significance.

## Results

### Roles of irisin, integrin αVβ5, and GRP78 in COPD pathogenesis

**Fig. 1:**
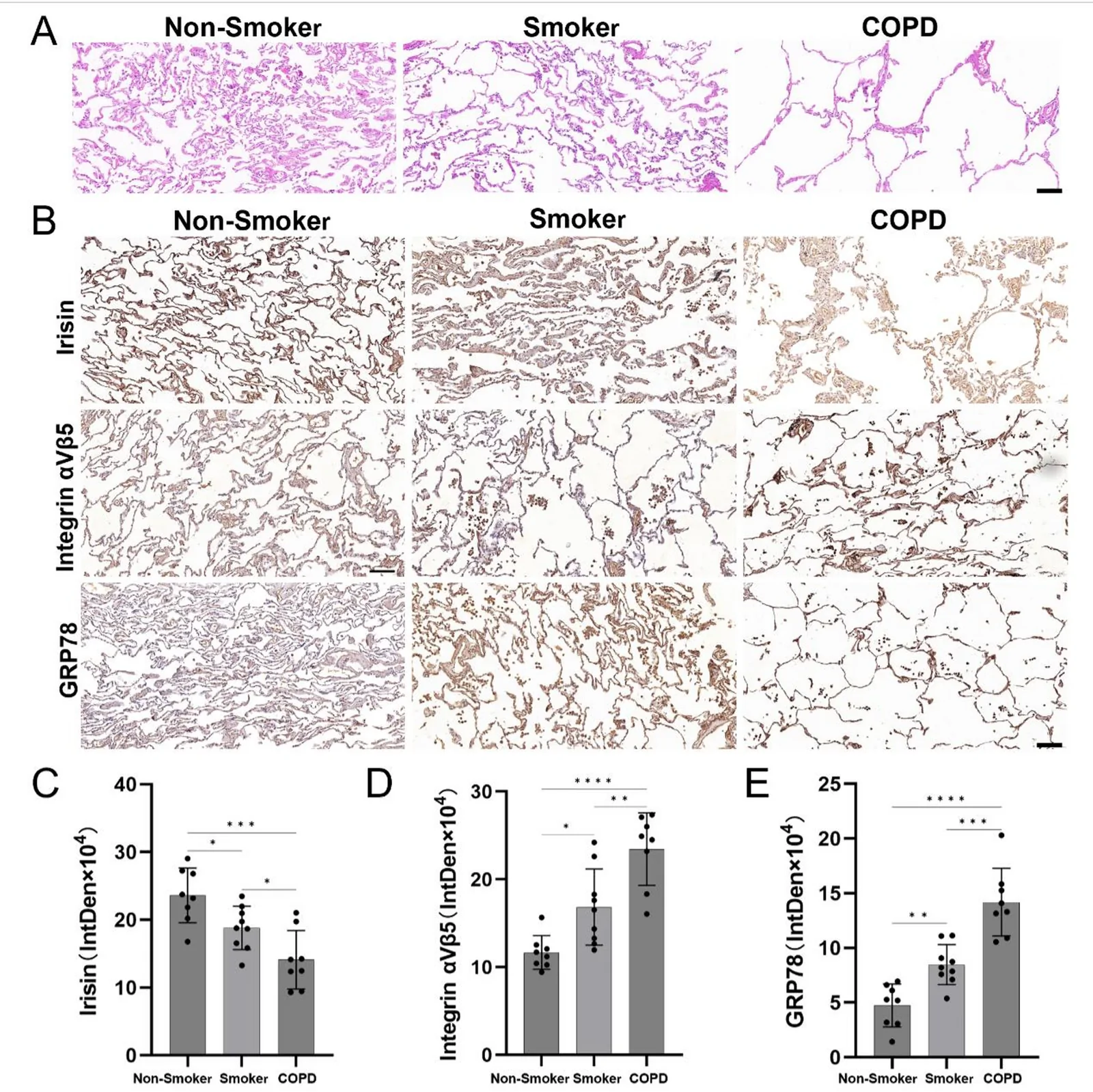
Roles of Irisin, Integrin αVβ5, and GRP78 in COPD Pathogenesis. **A** HE staining of human lung tissues. Scale bars, 100 μm. **B** IHC staining of Irisin, Integrin αVβ5, and GRP78 in lung tissues from Non-Smokers, Smokers and COPD patients. Scale bars, 100 μm. **C**, **D**, **E** IHC results were scored by the integrated density (IntDen). Data are presented as Mean ± S.D. (*n* = 8 or 9). *P < 0.05, **P < 0.01, ***P < 0.001, ****P < 0.0001 between groups

All participants underwent HE staining to assess lung histopathology. The nonsmoker group displayed an intact alveolar architecture with minimal inflammatory infiltration (Fig. 1A). In contrast, the smoker group exhibited alveolar wall destruction, alveolar enlargement, and mild infiltration of macrophages and other inflammatory cells. These changes were markedly exacerbated in the COPD group, which demonstrated a substantial reduction in alveolar number, thinning of the alveolar walls, significant alveolar enlargement, and the merging of alveolar spaces into enlarged cystic formations.

Quantitative immunohistochemical analysis revealed a progressive decrease in irisin expression across the groups, with the COPD cohort displaying the lowest irisin immunoreactivity (pale brown), which was primarily localized within alveolar epithelial cells and macrophages (Fig. 1B, C). Conversely, integrin αVβ5 expression increased in an exposure-dependent manner, ranging from minimal staining in non-smokers (pale brown) to moderate upregulation in smokers (brown) and robust elevation in COPD patients (dark brown) (Fig. 1B, D). GRP78 is a classic marker of ERS, and its expression is upregulated during stress [48]. Therefore, GRP78 staining was performed. Consistently, the expression of the canonical ERS marker GRP78 was significantly greater in the COPD cohort (dark brown) than in both the non-smoker and smoker groups (Fig. 1B, E) and was predominantly localized within alveolar epithelial cells and macrophages. Collectively, these findings indicate that smoking attenuates irisin expression while concurrently increasing integrin αVβ5 and GRP78 levels. The gradation of these molecular changes—from non-smokers to smokers to COPD patients—underscores the potential involvement of irisin deficiency, integrin αVβ5 activation, and ERS in the pathogenesis and progression of COPD.

### Differential expression of ERS-related genes in COPD

**Fig. 2:**
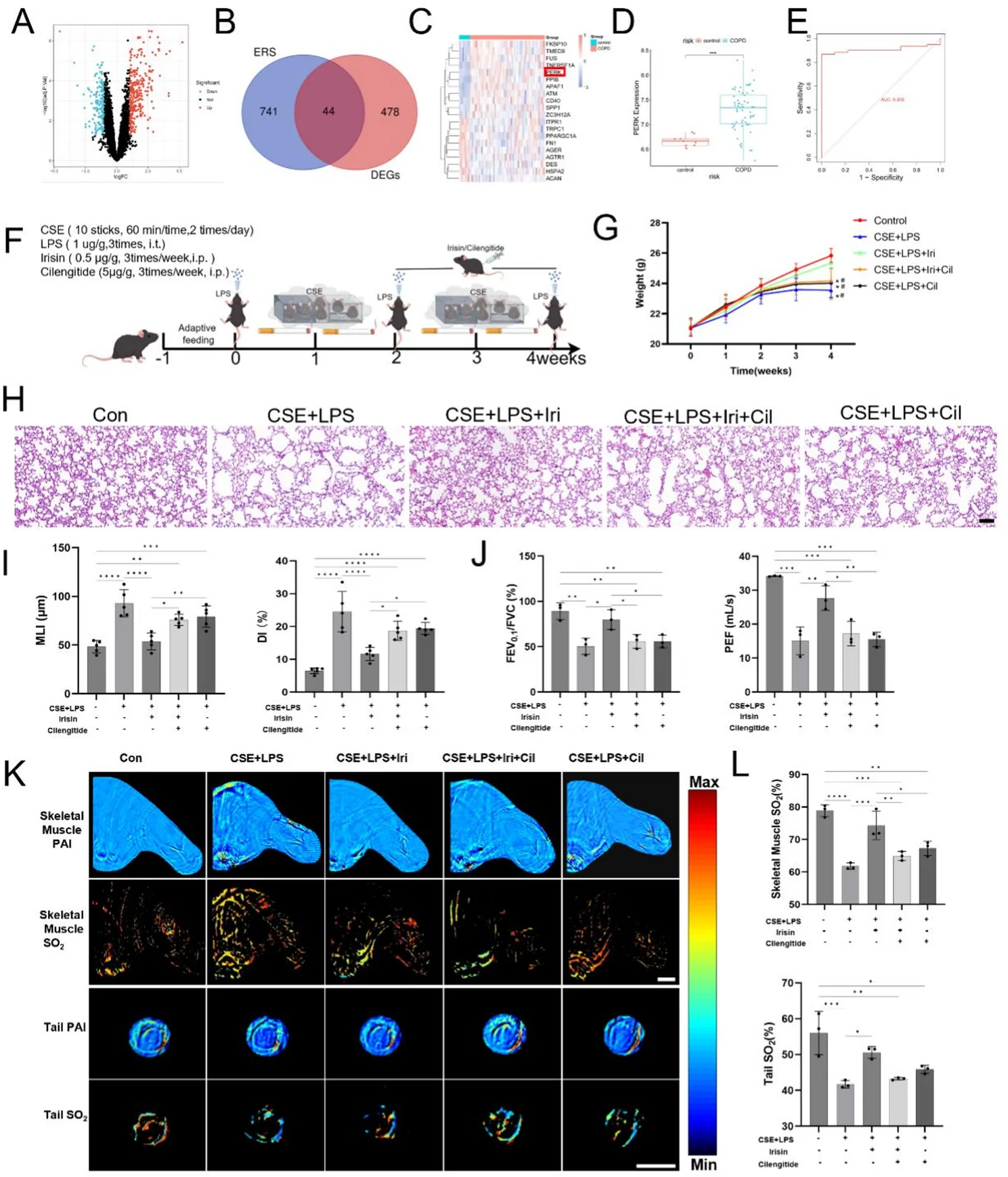
Identification of ERS-related hub genes and inhibition of integrin αvβ5 attenuates irisin-mediated protective effect in CSE + LPS treated mice. **A** Volcano plots of GSE38974 and GSE69818. **B** Venn diagram of DEGs and ERSs. **C** Heatmap of ERS-Related DEGs. **D** Box Plot of PERK expression in control and COPD groups. **E** The ROC curve of PERK. **F** Establishment of a mouse model by CSE + LPS. **G** Mice weight changes were examined. **H** Mice lung structural changes were examined by HE staining. (*n* = 5). Scale bars, 100 μm. **I** Pathological scores of lungs structural injury by MLI and DI. (*n* = 5) (**J**) Lung function measurement of lung structural injury by FEV0.1/FVC and PEF. (*n* = 3) (**K**) Photoacoustic imaging of skeletal muscle and tail. (*n* = 3). Scale bars, 3 mm. **L** Oxygen saturation of skeletal muscle and tail. Data are presented as Mean ± S.D. (*n* = 5). *P < 0.05, **P < 0.01, ***P < 0.001 between groups

Previous studies have confirmed that the PERK-associated ERS pathway modulates pulmonary inflammation and lung injury. We therefore utilized GEO datasets (GSE38974 and GSE69818) to provide relevant evidence. We identified 522 differentially expressed genes (DEGs) between the control and COPD groups across the two datasets. These DEGs were visualized using a volcano plot (Fig. 2A). We then intersected these DEGs with a known ER stress–related gene set and obtained 44 overlapping ERS-related DEGs, all of which were significantly dysregulated in the COPD samples (Fig. 2B–C). PERK, a canonical marker of the ERS pathway, was among these overlapping genes and was significantly upregulated in COPD patients (Fig. 2D). ROC curve analysis revealed excellent diagnostic performance with an AUC of 0.909 (Fig. 2E). Given that PERK is a core and well-established mediator of the ER stress response, we focused on the PERK/ATF4/CHOP signalling pathway for subsequent mechanistic investigations.

### The suppression of integrin αvβ5 attenuates irisin-mediated amelioration of lung injury and dysfunction in CSE + LPS-treated mice

**Fig. 3:**
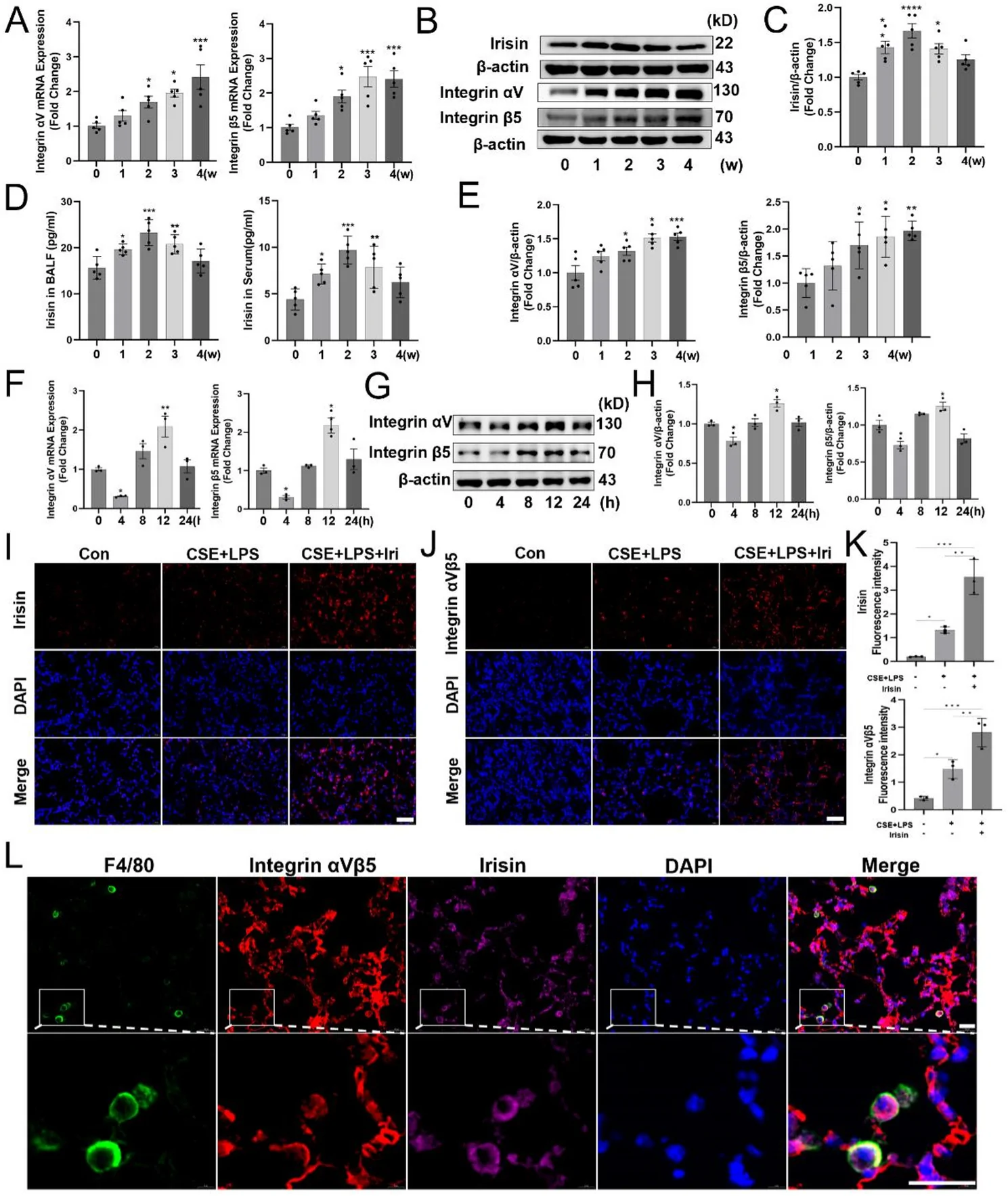
Expression of irisin and integrin αVβ5 in CSE+LPS treated mice and MH-S cells. **A** qRT-PCR analysis of integrin αV and β5 mRNA expression in vivo. (*n* = 5) (**B**, **C**, **E**) Western blot analysis of irisin, integrin αV and β5 expression in vivo. (*n* = 5). **D** ELISA of irisin in BALF and serum. (*n* = 5). **F** qRT-PCR analysis of integrin αV and β5 mRNA expression in vitro. (*n* = 3). **G**, **H** Western blot analysis of irisin, integrin αV and β5 expression in vitro. (*n* = 3). **I**, **J**, **K** Immunofluorescence staining of irisin and integrin αVβ5 in lung tissues. Scale bars, 50 μm. (*n* = 3). **L** Triple-label immunofluorescence confocal microscopy for F4/80 (green), integrin αVβ5 (red), and irisin (magenta) in lung tissue. Scale bars, 20 μm. (*n* = 2). *P < 0.05, **P < 0.01, ***P < 0.001, ****P < 0.0001 between groups

A mouse model of AECOPD was successfully induced by CSE + LPS exposure (Fig. 2F). CSE + LPS treatment caused significant weight loss, pronounced emphysematous changes (alveolar wall thinning and disruption, increased MLI and DI, and decreased alveolar number), impaired pulmonary function (reduced FEV0.1/FVC and elevated PEF), and decreased skeletal muscle and tail oxygen saturation (SO2), whereas control mice exhibited gradual weight gain (Fig. 3G–L). Exogenous irisin effectively reversed these CSE + LPS-induced abnormalities. However, these beneficial effects of irisin were abolished by coadministration of the integrin αvβ5 inhibitor cilengitide. Cilengitide alone had no significant effect on CSE + LPS-treated mice.

### Temporal dynamics of irisin and integrin αvβ5 expression in CSE + LPS-treated mice and MH-S cells

To investigate the temporal changes in irisin and αVβ5 expression in vivo following CSE + LPS treatment, we conducted qRT-PCR, Western blotting, and ELISA (Fig. 3A–E). The expression levels were assessed at 0 (control), 1, 2, 3, and 4 weeks after CSE and LPS exposure. Relative to those in the control group, the expression levels of integrin αV and β5 were markedly upregulated, peaking at 4 weeks. Conversely, irisin expression gradually increased, peaked at 2 weeks, and subsequently decreased at weeks 3 and 4. The temporal expression profile of integrin αVβ5 was evaluated at 0 (control), 4, 8, 12, and 24 h in vitro. Compared with that in the control group, the expression of integrin αV and β5 significantly decreased at 4 h, increased at 8 h, peaked at 12 h, and subsequently decreased at 24 h (Fig. 3F-H). Consequently, the 12-hour time point was selected for subsequent experiments.

Immunofluorescence staining revealed significant upregulation of irisin expression in vivo. Exogenous supplementation with irisin further increased irisin and integrin αVβ5 expression (Fig. 3I–K). To investigate the potential interaction between irisin and integrin αVβ5 in lung macrophages, triple immunofluorescence confocal microscopy was performed, to label F4/80 (green), integrin αVβ5 (red), and irisin (magenta). We observed overlapping fluorescence, indicating the pronounced colocalization of irisin and integrin αVβ5 in mouse lung macrophages (Fig. 3L).

### Molecular dynamics simulations elucidate the binding mechanism between irisin and integrin αvβ5

**Fig. 4:**
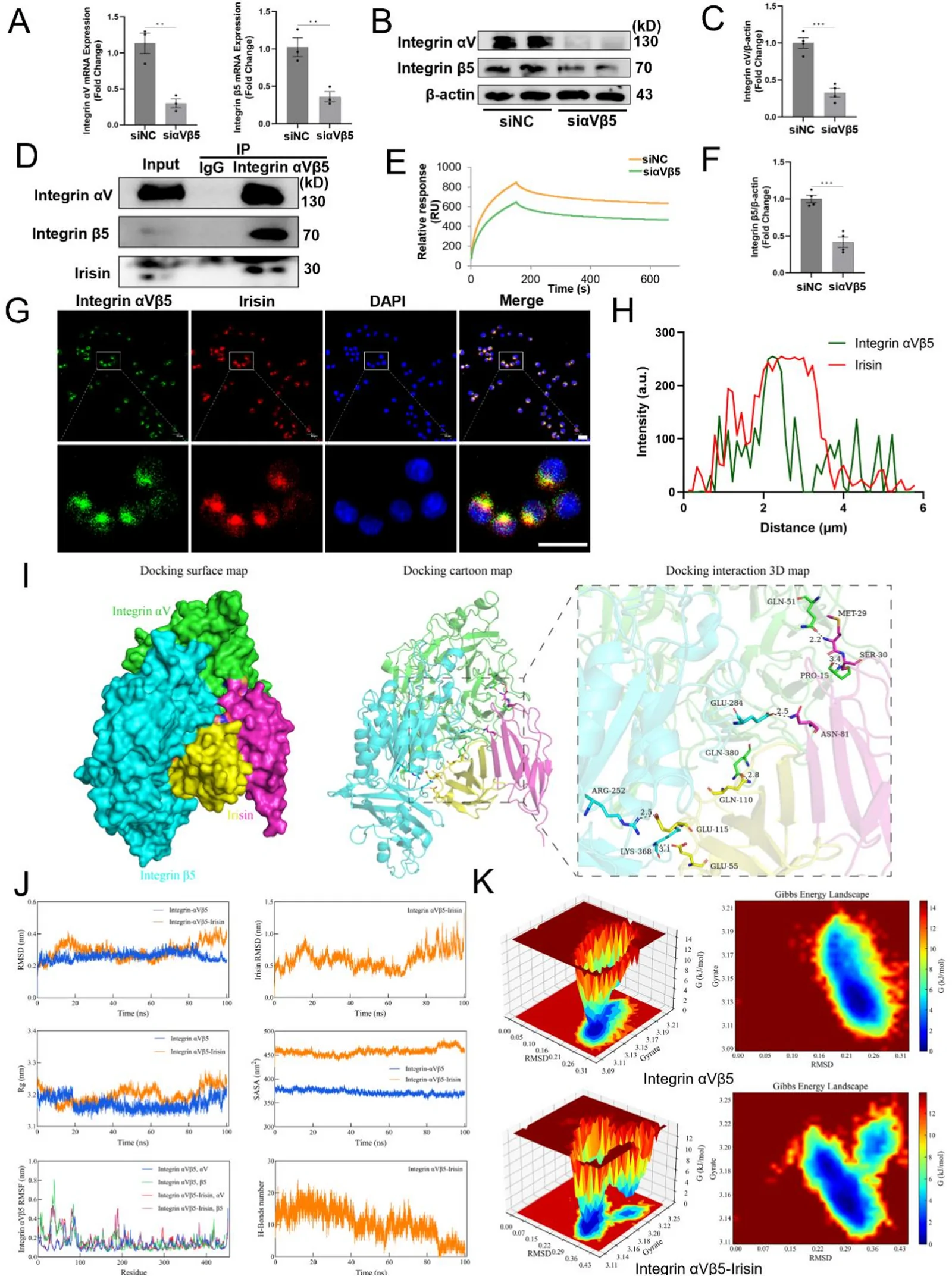
The binding mechanism of irisin with integrin αvβ5. **A** qRT-PCR analysis of integrin αV and β5 mRNA expression. (*n* = 3). **B**, **C**, **F** Western blot analysis of integrin αV and β5 expression. (*n* = 3). **D** Co-IP of irisin with integrin αV and β5. **E** SPR analysis of the binding affinity between recombinant irisin and siNC or siαVβ5 cells. **G**, **H** Immunofluorescence staining of integrin αVβ5 (green), irisin (red) in MH-S cells. Scale bars, 50 μm. **I** Molecular docking of irisin with integrin αVβ5. **J** MD simulation of the irisin-integrin αVβ5 complex over 100 ns. **K** Gibbs free energy landscape of irisin with integrin αVβ5. **P < 0.01, ***P < 0.001 between groups

To investigate the mechanism underlying the interaction between irisin and integrin αVβ5, we knocked down integrin αVβ5 using specific siRNAs targeting integrin αV (siαV) and β5 (siβ5) (Fig. 4A). Co-transfection of MH-S cells with siαV and siβ5 significantly reduced αV and β5 protein expression (Fig. 4B, C, F). Co-IP analysis verified the direct interaction with integrin αVβ5 in irisin-treated macrophages (Fig. 4D). SPR revealed that compared with negative control cells, αVβ5-knockdown cells had fewer resonance units (RUs) for recombinant irisin and reduced binding capacity (Fig. 4E). Immunofluorescence staining revealed obvious irisin–αVβ5 colocalization in treated MH-S cells (Fig. 4G, H).

Molecular docking and 100 ns MD simulation were used to explore the atomic-level interaction mode. Rigid docking of integrin αVβ5 and irisin via GRAMM revealed that irisin was embedded in the integrin αVβ5 subunit interface (good geometric complementarity). Docking revealed multiple stable hydrogen bonds (2.2–3.1 Å) between irisin and αVβ5, involving key residues (PRO-15, MET-29, SER-30, ASN-81, GLU-284, GLN-380, ARG-252, GLU-115, LYS-368, and GLU-55) (Fig. 4I).

MD simulation was used to analyse the RMSD, RMSF, Rg, SASA, and number of hydrogen bonds (Fig. 4J). The free αVβ5 RMSD stabilized at 0.2–0.25 nm; the integrin αVβ5–irisin complex had a slightly higher RMSD (0.2–0.42 nm, acceptable < 0.5 nm). The RMSD of irisin fluctuated between 0.5 and 1.4 nm, suggesting conformational flexibility and induced-fit binding. Both free αVβ5 and the complex had Rg fluctuations (3.15–3.25 nm), indicating structural stability. The integrin αVβ5 SASA was lower than that of the complex (increased surface area post-binding). RMSF showed higher αV subunit fluctuation (binding involvement) vs. stable β5 (anchoring role). The number of hydrogen bonds decreased from ~ 25 to 10–15 and stabilized at 0–5 (80–100 ns), confirming complex stability. The Gibbs free energy landscape revealed that isolated αVβ5 had one deep, narrow energy well (high stability/rigidity) (Fig. 4K). After irisin binding, two distinct wells appeared with broader RMSD/Rg distributions, indicating increased αVβ5 flexibility and rearrangement, facilitating adaptation to signal transduction/cell adhesion functions. MM/PBSA analysis was used to quantify the binding free energy (Table 3): total Gibbs free energy (ΔGbind) = -19.90 kcal/mol (thermodynamic stability). Van der Waals forces (ΔEvdW = -142.10 kcal/mol) and non-polar solvation energy (ΔGsurf = -18.96 kcal/mol) were key contributors. Large electrostatic interactions (ΔEelec = 1111.51 kcal/mol) were counteracted by solvent shielding (ΔGGB = -970.35 kcal/mol). Irisin- integrin αVβ5 binding is driven by hydrophobic forces and local polarity adjustments (induced-fit stable interface), supporting molecular recognition studies and drug design.

**Table 3:** The average binding free energy of the complex is calculated by the MM/PBSA method(kcal/mol)

Energy contributions	Integrin αVβ5-Irisin
ΔE_vdW_	-142.10
ΔE_elec_	1111.51
ΔG_GB_	-970.35
ΔG_surf_	-18.96
ΔG_gas_	969.41
ΔG_solv_	-989.31
ΔG_bind_	-19.90

These results contribute to the specific recognition and stable binding between irisin and integrin αVβ5, which is consistent with our previous Co-IP, SPR, and immunofluorescence results demonstrating their direct interaction in macrophages. These findings lay a critical foundation for further verification of the irisin–integrin αVβ5 interaction and its downstream signalling pathways.

### Irisin inhibits inflammation through integrin αvβ5 in CSE + LPS-treated mice and MH-S cells

**Fig. 5:**
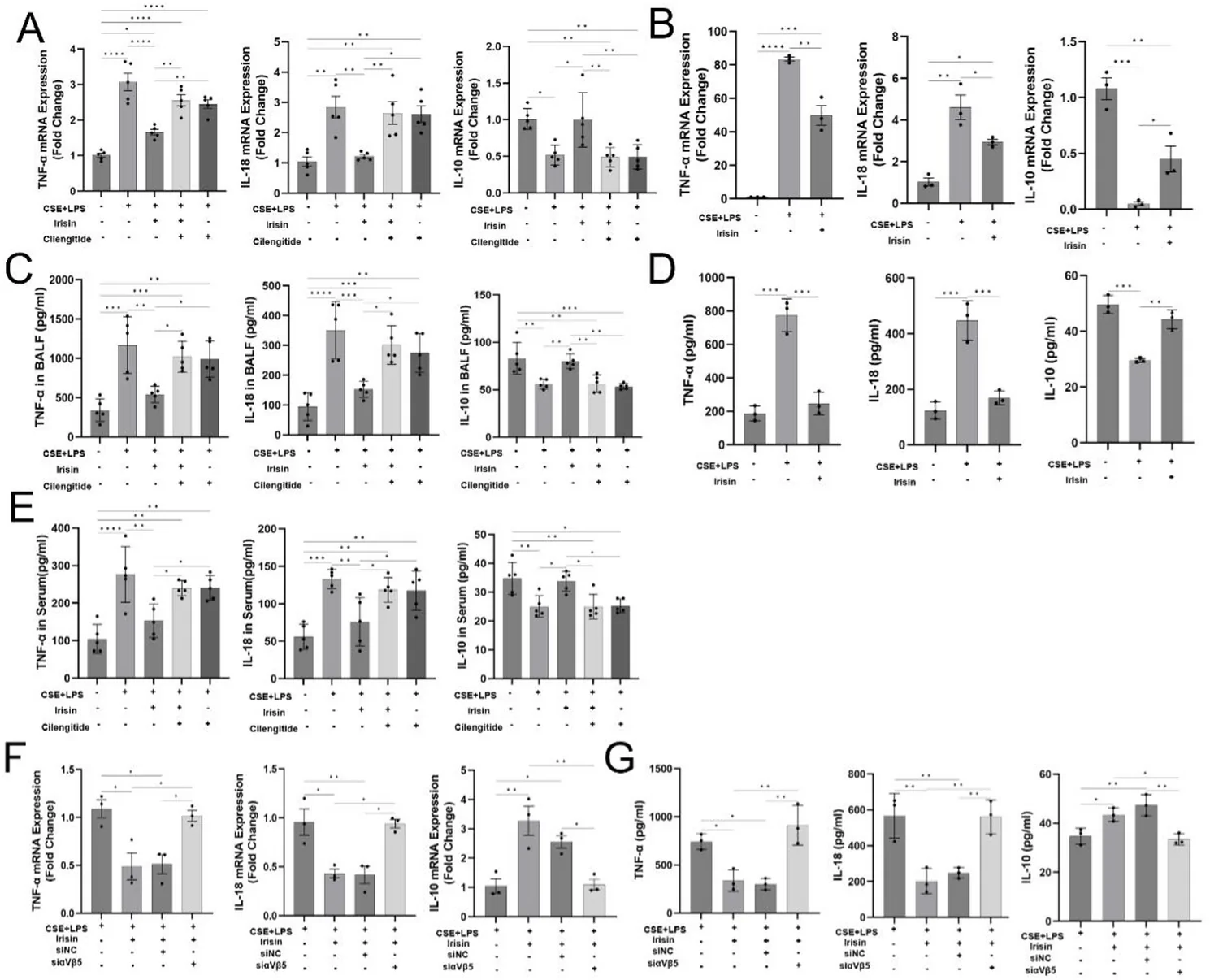
Irisin inhibits CSE + LPS-induced inflammation through integrin αvβ5. **A** qRT-PCR analysis of TNF-α, IL-18 and IL-10 mRNA expression in lung tissue. (*n* = 5). **C**, **E** ELISA of TNF-α, IL-18 and IL-10 in BALF and serum. (*n* = 5). **B**, **F** qRT-PCR analysis of TNF-α, IL-18 and IL-10 mRNA expression in MH-S cells. (*n* = 3). **D**, **G** ELISA of TNF-α, IL-18 and IL-10 in cellular supernatant. (*n* = 3). *P < 0.05, **P < 0.01, ***P < 0.001, ****P < 0.0001 between groups

Having established the interaction between irisin and integrin αVβ5, we next investigated whether integrin αVβ5 was required for the anti-inflammatory effects of irisin in the CSE + LPS-treated mouse and cell models. In vivo, inflammatory cytokine profiling via qRT‒PCR and ELISA revealed that the CSE + LPS-induced increase in TNF-α and IL-18 levels concurrently restored the suppressed IL-10 levels. Cilengitide effectively counteracts the anti-inflammatory effects of irisin. (Fig. 5A, C, E).

In vitro, qRT-PCR analysis of cellular mRNA and ELISA quantification of cell supernatants demonstrated that CSE + LPS exposure markedly elevated TNF-α and IL-18 levels while suppressing IL-10 levels. Irisin administration markedly suppressed the production of TNF-α and IL-18 and restored IL-10 levels compared with those in the CSE + LPS group (Fig. 5B, D). Importantly, knockdown of integrin αVβ5 abolished these effects (Fig. 5F, G). 

### Irisin maintains ER homeostasis and activates AMPK through integrin αvβ5 in CSE + LPS-treated mice and MH-S cells

**Fig. 6:**
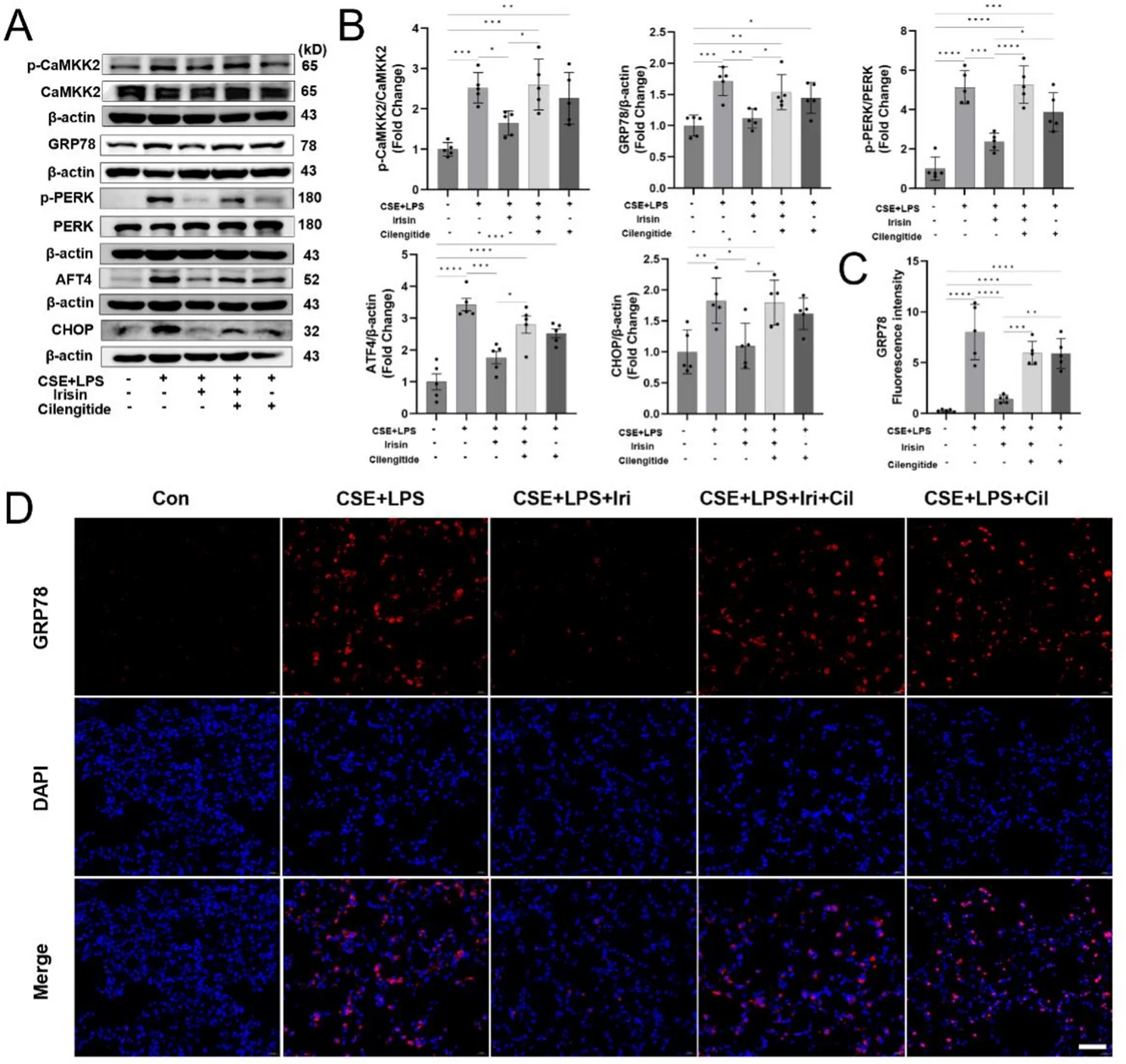
Inhibition of integrin αvβ5 reverses Irisin-mediated alleviation of ERS in CSE + LPS-treated mice. **A**, **B** Western blot analysis of ERS-related proteins expression in vivo. (*n* = 5). **C**, **D** Immunofluorescence staining of GRP78 (red) in lung tissues. (*n* = 5). Scale bars, 50 μm. *P < 0.05, **P < 0.01, ***P < 0.001, ****P < 0.0001 between groups

After establishing the integrin αVβ5-dependent protective and anti-inflammatory effects of irisin, we further investigated the downstream molecular responses associated with this receptor, focusing on ER homeostasis and AMPK activity. To further elucidate the mechanisms underlying the efficacy of irisin and the role of integrin αVβ5, the activity of the PERK pathway—which is key to ERS—was assessed by Western blotting in vivo. Compared with the control group, the CSE + LPS group exhibited marked upregulation of p-CaMKK2, GRP78, p-PERK, ATF4, and CHOP, which is indicative of ERS. Administration of exogenous irisin attenuated the expression of these proteins. Notably, cotreatment with cilengitide abrogated the protective effects of irisin (Fig. 6A, B). Consistent with the Western blot findings, the IF imaging results for GRP78 in lung tissue corroborated these results (Fig. 6C, D).

**Fig. 7:**
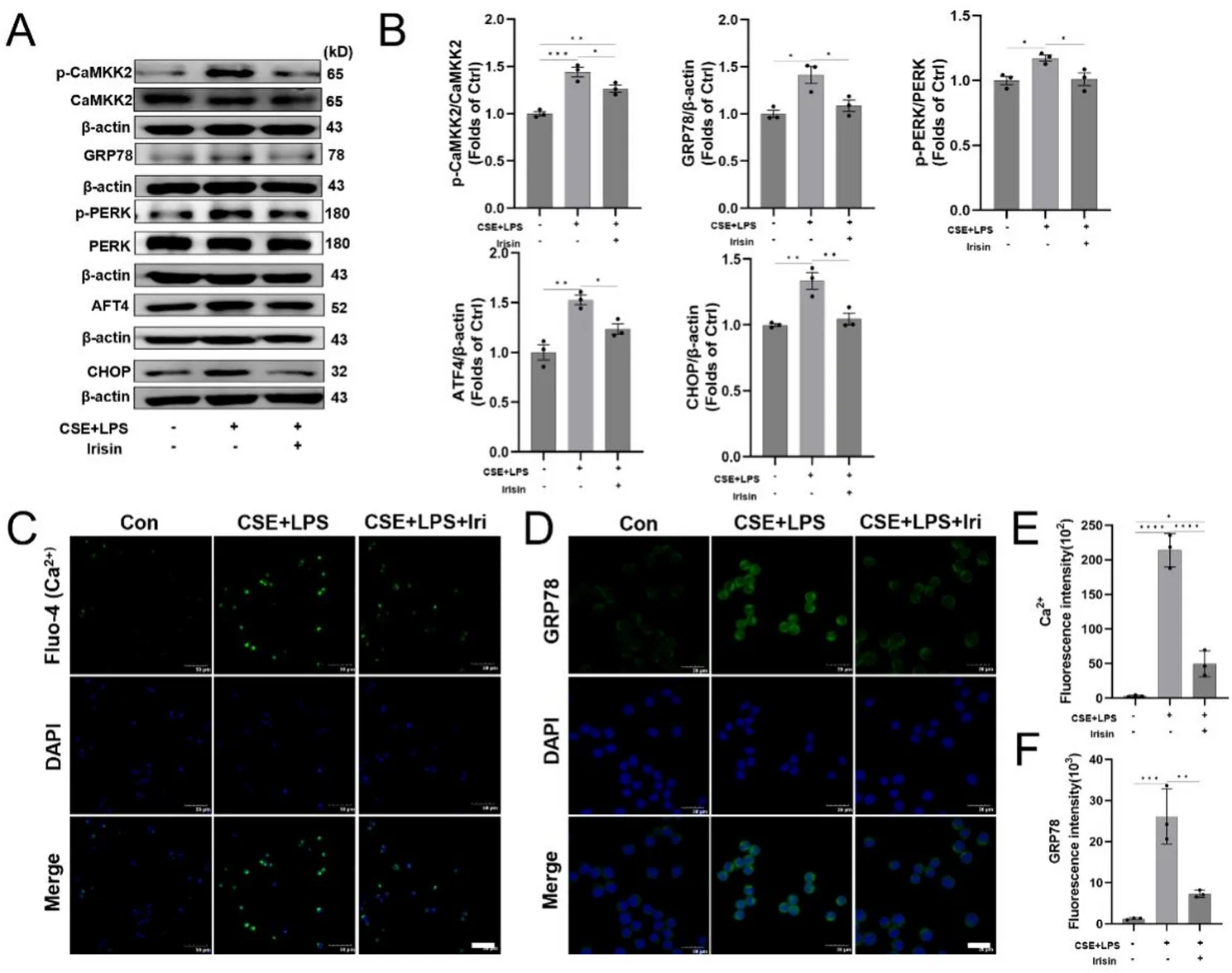
Irisin restores ER homeostasis in CSE + LPS-treated MH-S cells. **A**, **B** Western blot analysis of ERS-related proteins expression. (*n* = 3). **C**, **E** Immunofluorescence staining of Fluo-4 (Ca²⁺) (green). Scale bars, 50 μm. (*n* = 3). **D**, **F** Immunofluorescence staining of GRP78 (green). Scale bars, 20 μm. (*n* = 3). *P < 0.05, **P < 0.01, ***P < 0.001, ****P < 0.0001 between groups

At 12 h post-CSE + LPS treatment, Western blot analysis revealed a marked increase in the expression levels of p-CaMKK2, GRP78, p-PERK, ATF4, and CHOP in MH-S cells. Exogenous irisin treatment effectively attenuated the expression of these proteins (Fig. 7A, B). Immunofluorescence staining demonstrated markedly higher fluorescence intensities of Fluo-4 (Ca²⁺) and GRP78 in the CSE + LPS group han in the control group. Notably, irisin treatment significantly reduced the fluorescence signals of both Ca²⁺ and GRP78 (Fig. 7C–F).

**Fig. 8:**
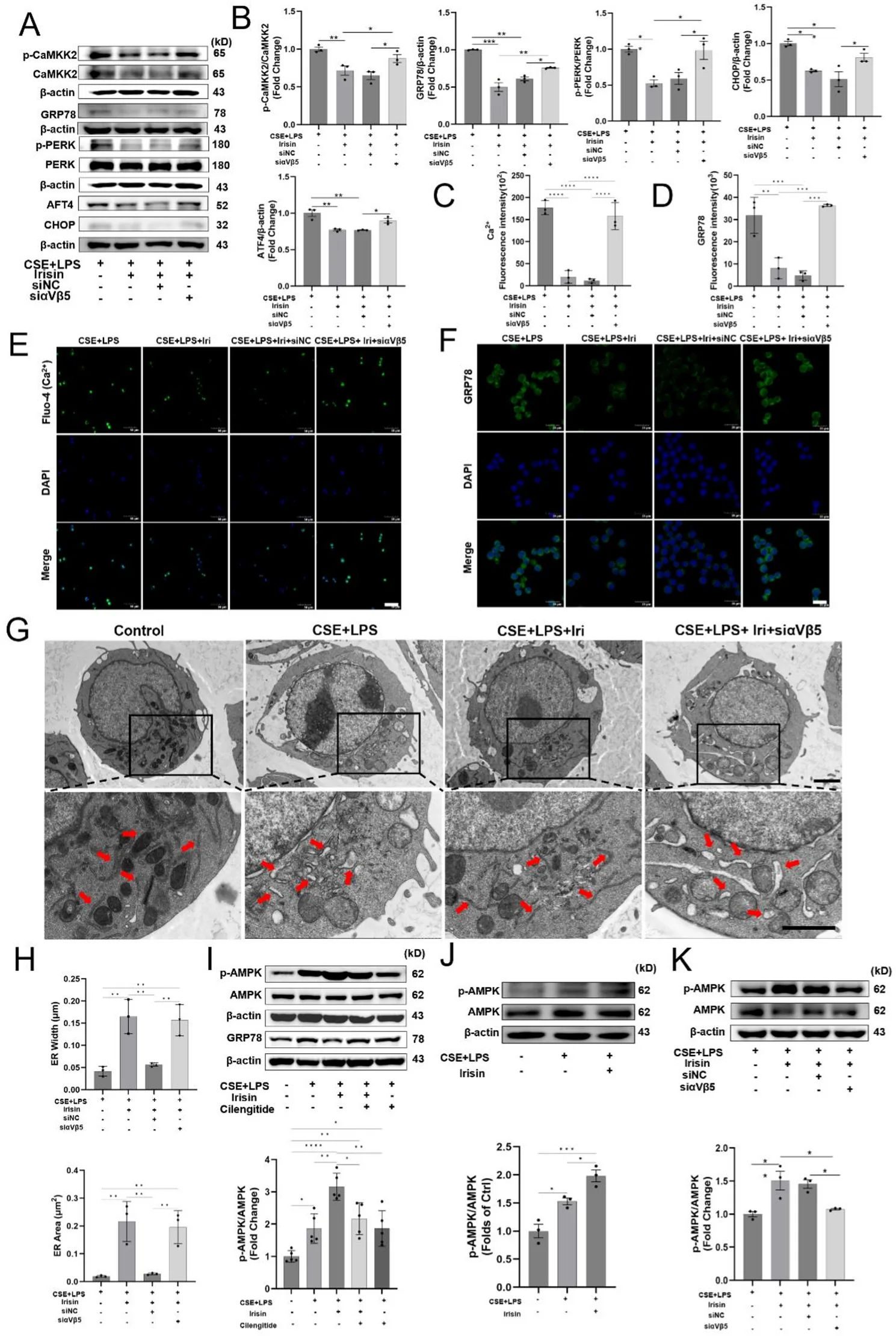
Irisin maintains ER homeostasis and activates AMPK through integrin αvβ5 in CSE + LPS-treated mice and MH‑S cells. **A**, **B** Western blot analysis of ERS-related proteins expression. (*n* = 3). **C**, **E** Immunofluorescence staining of Fluo-4 (Ca²⁺) (green). Scale bars, 50 μm. (*n* = 3). **D**, **F** Immunofluorescence staining of GRP78 (green). Scale bars, 20 μm. (*n* = 3). **G**, **H** TEM images of MH-S cells in different treatment groups. The red arrow represents ER. Scale bars, 2 μm. (*n* = 3). **I** Western blot analysis of AMPK proteins expression in vivo. (*n* = 5). **J**, **K** Western blot analysis of AMPK proteins expression in vitro. (*n* = 3). *P < 0.05, **P < 0.01, ***P < 0.001, ****P < 0.0001 between groups

To further confirm that irisin exerts its protective effects through integrin αVβ5, we knocked down integrin αVβ5 in MH-S cells. The Western blotting results demonstrated that, compared with the CSE + LPS group, irisin treatment significantly decreased the expression levels of p-CaMKK2, GRP78, p-PERK, ATF4, and CHOP. Importantly, knockdown of integrin αVβ5 abolished these effects: the expression of p-CaMKK2, GRP78, p-PERK, ATF4, and CHOP was restored to levels comparable to those in the CSE + LPS group (Fig. 8A, B). Consistent with the Western blot results, the Ca²⁺ fluorescence intensity was significantly lower in irisin-treated cells than in CSE + LPS -treated cells, but this reduction was reversed by integrin αVβ5 knockdown, with the Ca²⁺ intensity returning to a level similar to that in the CSE + LPS group (Fig. 8C, E). Similarly, the immunofluorescence staining of GRP78 was consistent with the Western blot data (Fig. 8D, F).

TEM was performed to visualize the ultrastructural alterations of the ER under different treatments. Within the control group, the ER displayed a typical, densely organized morphology. Stimulation with CSE + LPS induced prominent ER swelling, dilation, and structural disorganization (red arrows), indicating severe ER stress. Treatment with irisin (CSE + LPS+Iri) markedly alleviated these morphological abnormalities, preserving normal ER structure. However, knockdown of integrin αVβ5 (CSE + LPS+Iri+siαVβ5) abolished the effects of irisin, as demonstrated by the reappearance of swollen and disorganized ER, which was comparable to that in the CSE + LPS group (Fig. 8G, H).

In addition to its effects on ER homeostasis, we examined whether irisin regulated AMPK activity as another downstream response dependent on integrin αVβ5. In vivo, CSE + LPS exposure increased AMPK phosphorylation, and irisin treatment further enhanced p-AMPK levels. This additional increase was abolished by cotreatment with cilengitide (Fig. 8I). Similarly, in MH-S cells, p-AMPK levels were elevated after CSE + LPS exposure and were further increased by irisin treatment (Fig. 8J). Knockdown of integrin αVβ5 suppressed the irisin-induced increase in p-AMPK (Fig. 8K). Together with the ER-homeostasis findings, these results indicate that integrin αVβ5 is required for both the maintenance of ER homeostasis and the enhancement of AMPK activity induced by irisin. On the basis of the present data, these effects are interpreted as parallel downstream responses of integrin αVβ5 signaling, and a direct causal relationship between ER homeostasis and AMPK activation cannot yet be established.

Collectively, integrin αVβ5 inhibition or knockdown abolished both the effects of irisin on ER-homeostasis-related markers and its enhancement of AMPK phosphorylation. These findings support integrin αVβ5 as a key receptor linking irisin to two coordinated downstream responses: maintenance of ER homeostasis and enhancement of AMPK activity.

### Irisin attenuates CSE + LPS-induced inflammatory response by maintaining ER homeostasis in CSE + LPS-treated MH-S cells

**Fig. 9:**
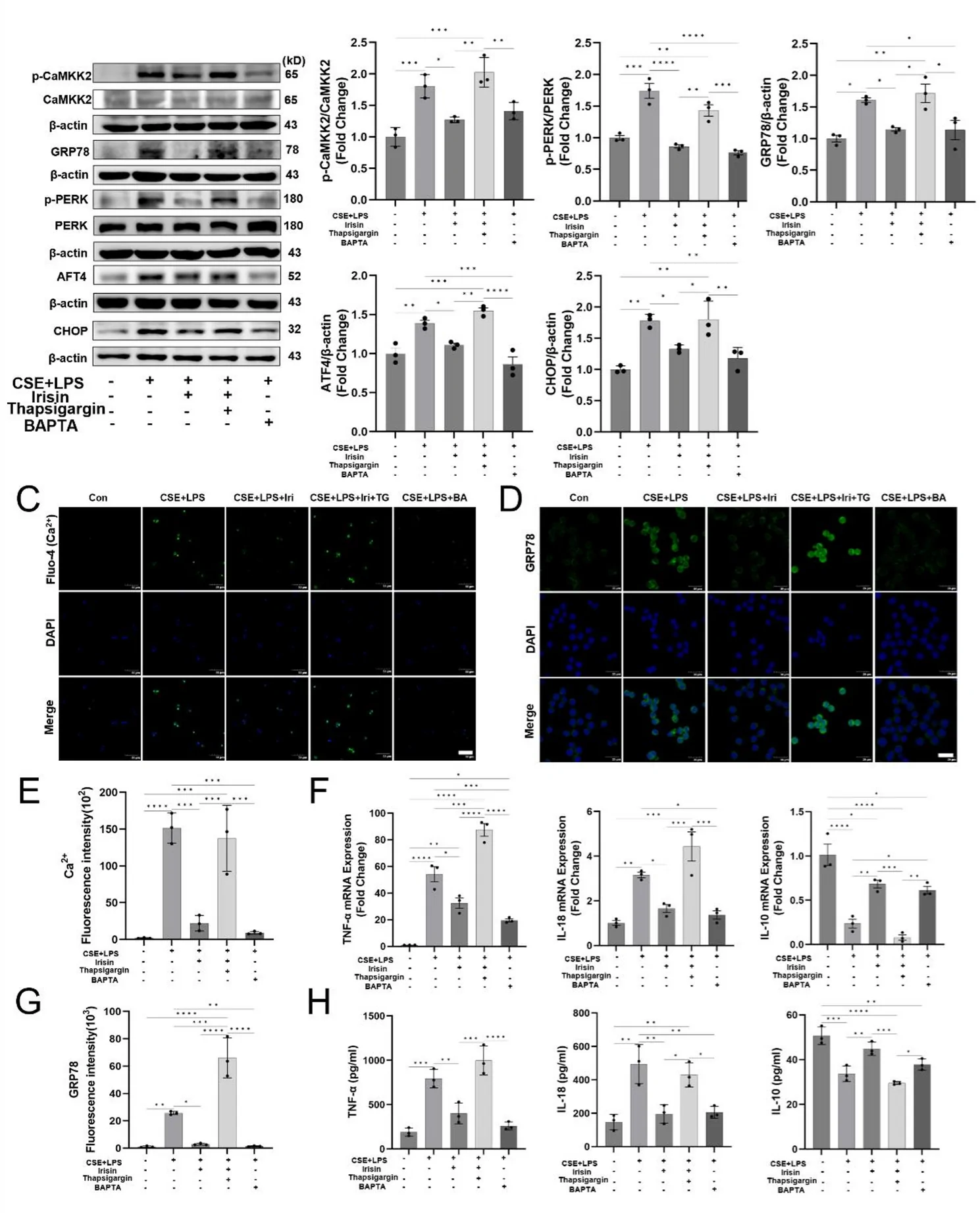
Irisin attenuates inflammatory response in CSE + LPS-treated MH-S cells by maintaining ER homeostasis. **A**, **B** Western blot analysis of ERS-related proteins expression. (*n* = 3). **C**, **E** Immunofluorescence staining of Fluo-4 (Ca²⁺) (green). Scale bars, 50 μm. (*n* = 3). **D**, **G** Immunofluorescence staining of GRP78 (green). Scale bars, 20 μm. (*n* = 3). **F** qRT-PCR analysis of TNF-α, IL-18 and IL-10 mRNA expression. (*n* = 3). (H) ELISA of TNF-α, IL-18 and IL-10 in cellular supernatant. (*n* = 3). *P < 0.05, **P < 0.01, ***P < 0.001, ****P < 0.0001 between groups

To further examine whether maintenance of ER homeostasis contributes to the anti-inflammatory effects of irisin, MH-S cells were treated with the ER stress inducer Thapsigargin or the intracellular Ca²⁺ chelator BAPTA. hapsigargin induces ER stress by inhibiting the sarco/endoplasmic reticulum Ca²⁺-ATPase [49], whereas BAPTA reduces cytosolic Ca²⁺ accumulation through intracellular Ca²⁺ chelation [50]. Western blot analysis demonstrated that CSE + LPS treatment markedly increased the phosphorylation of CaMKK2, PERK, GRP78, ATF4, and CHOP. Irisin treatment effectively reversed these effects, reducing the expression of p-CaMKK2, GRP78, p-PERK, ATF4, and CHOP. Importantly, the protective effects were abolished by Thapsigargin. Conversely, the effects of BAPTA are consistent with those of irisin, which significantly reduced the protein expression of ERS-related proteins (Fig. 9A, B). CSE + LPS treatment significantly increased the intracellular Ca²⁺ and GRP78 fluorescence intensity, whereas irisin treatment can significantly reduce this intensity; Thapsigargin reversed the effect of irisin, whereas BAPTA simulated the effect of irisin (Fig. 9C–E, G). After CSE + LPS treatment, the levels of TNF-α and IL-18 significantly increased, whereas the level of IL-10 was markedly decreased. Irisin treatment effectively reversed these inflammatory changes. However, Thapsigargin abolished the effects of irisin, whereas BAPTA reversed the protective effects of irisin (Fig. 9F, H). These results confirm that ER homeostasis is a critical upstream mediator of the protective effects of irisin against inflammation.

## Discussion

This research aims to elucidate the role and underlying mechanisms of irisin via integrin αVβ5 in COPD. We demonstrate that irisin acts through integrin αVβ5 to promote two coordinated protective responses in CSE + LPS-treated models: maintenance of ER homeostasis and activation of AMPK. These responses are associated with reduced emphysematous injury and inflammation, with modulation of the PERK signaling pathway contributing to the ER-homeostasis branch.

IHC analysis of human lung tissue revealed that the level of irisin was highest in non-smoking healthy individuals, decreased in smokers, and lowest in smoker with COPD. These results align with the findings reported in prior research concerning serum concentrations [9, 51, 52]. In contrast, the expression of integrin αVβ5 tended to increase in smokers with COPD, which is in line with the conclusion that smoking causes pulmonary fibrosis and airway remodelling in asthma/COPD, in which the expression of integrin αVβ5 is elevated [53, 54]. During the animal modelling process, irisin levels initially increased, peaked at 2 weeks, and then gradually decreased, with no significant difference at 4 weeks compared with those at the beginning of the modelling. In contrast, integrin αVβ5 expression tended to increase throughout the modelling process, and peaked at 4 weeks. The temporal trend of irisin in this study was significantly different from that in mouse models of intracerebral haemorrhage and glaucoma-induced neuroinflammation [55, 56], while the trend of integrin αVβ5 was similar. The inconsistent expression trends of irisin and integrin αVβ5 may result from multiple factors, including stage-dependent regulation, potential negative feedback between the two proteins, distinct upstream regulatory pathways, and differences in the microenvironment of human tissues and animal models. Further studies are needed to clarify these mechanisms.

In the present study, we employed a multifaceted experimental approach to demonstrate that ithe biological function of irisin is mediated through its interaction with integrin αVβ5. This observation aligns with prior research examining the irisin–integrin signalling pathway [7, 15, 16]. Using multiplex immunofluorescence, we confirmed the colocalization of irisin and integrin αVβ5 in lung macrophages within murine lung tissues and cultured cells. Co-IP assays further verified their physical interaction, and knockdown of αVβ5 attenuated cellular binding to recombinant irisin protein. When integrin αVβ5 was knocked down, the protective effect of irisin was lost. These findings confirm that integrin αVβ5 is a key receptor for signal transduction mediated by irisin.

The molecular mechanisms underlying this functional interplay were further elucidated using in rigid docking and MD simulations. Structural analyses revealed a clear division of labour between the integrin subunits: the αV subunit forms the primary binding interface via specific hydrogen bonds and hydrophobic contacts, whereas the β5 subunit mediates conformational changes and signal propagation. Our present computational results support the established model in which integrin αVβ5 exists in a bent, closed resting state under basal conditions. Upon binding to irisin, it switches to the extended, open active conformation, enabling high-affinity binding and downstream signalling [57, 58]. These findings are consistent with cryo-EM observations of three conformational states of integrin αVβ5 [15] and provide solid computational and theoretical structural support for this activation mechanism. This paradigm, in which the α subunit dictates ligand specificity and the β subunit drives signalling, is a well-characterized hallmark of the integrin superfamily [59]. Collectively, these data support a model in which irisin specifically engages αVβ5 to alleviate damage induced by CES + LPS. This study not only identifies integrin αVβ5 as a functional receptor for irisin but also provides a structural framework for understanding how this interaction orchestrates cellular protective signalling pathways.

After knockdown of integrin αVβ5 or intervention with Thapsigargin, the aforementioned protective effect of irisin disappeared, and the effects of BAPTA-AM were similar to those of irisin. These results confirmed that irisin exerts a protective effect by regulating calcium overload and ERS pathways through integrin αVβ5, thereby maintaining ER homeostasis. Some studies have reported that irisin can inhibit Ca²⁺-dependent ERS in vascular smooth muscle cells [60]. These findings align with the conclusions drawn in the present study that irisin exerts protective effects by maintaining ER homeostasis”. We used differential gene screening of the GEO database to identify significant differences in the expression of the PERK gene. It is known that the PERK pathway is a classic signalling pathway of ERS [61, 62]. This suggests that it may be involved in the protective effect mediated by irisin. Further in-depth mechanism verification confirmed that irisin can further enhance the effect of ERS by regulating the PERK/ATF4/CHOP signalling pathway and alleviating pulmonary emphysema and inflammatory responses, thereby completing the downstream molecular regulatory network of the irisin–integrin αVβ5 axis and providing a more in-depth experimental basis for this protective mechanism.

Our findings, corroborated by those of previous studies [56, 63, 64], indicate that irisin exerts protective effects through integrin αVβ5-mediated activation of the AMPK signalling. Furthermore, our previous research has revealed that irisin modulates lung autophagy induced by CSE and LPS through the activation of the AMPK-Beclin1 signalling axis, thereby alleviating the inflammatory response [14]. Collectively, these data reveal a comprehensive regulatory cascade in which irisin, via integrin αVβ5, stimulates AMPK-Beclin1 signalling to modulate autophagy in the context of CSE + LPS exposure. This integrated pathway not only expands the downstream molecular network of the irisin–integrin αVβ5 axis but also seamlessly bridges our current findings with our previous work.

This study demonstrated that irisin exerts its effects by targeting integrin αVβ5. This study has several limitations. Cilengitide lacks absolute target specificity and tends to cause off-target effects, which may affect the results of the analysis. The quantities of human tissues and experimental animals in this study are limited, leading to insufficient sample representativeness. In addition, this study explored only basic mechanisms and did not evaluate drug efficacy or safety. Further studies should adopt gene targeting methods to avoid off-target interference, expand the sample size and verify the relevant mechanisms.

## Conclusion

**Fig. 10:**
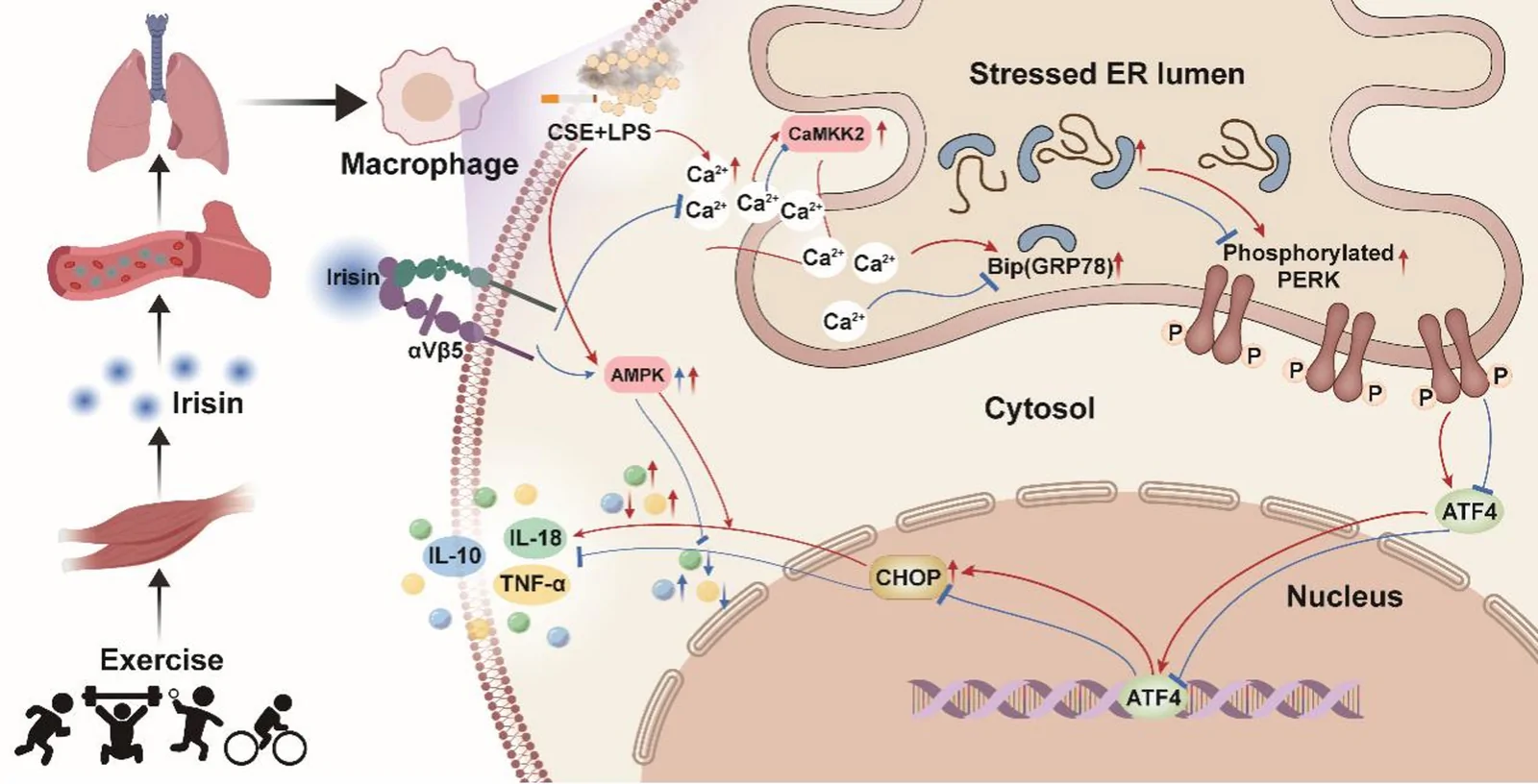
Hypothesized schematic presentation of the protective mechanism of irisin in CSE + LPS-treated model. Irisin, through integrin αVβ5, concurrently maintains ER homeostasis, and enhances AMPK activity, thereby alleviating emphysema and inflammatory responses induced by CSE combined with LPS. Red lines indicate pathological signaling cascades triggered by CSE + LPS, whereas blue lines indicate protective signaling cascades initiated by irisin. Solid arrows indicate activation, whereas flat bar-ended lines indicate inhibition. Upward (↑) and downward (↓) arrows indicate increased and decreased expression levels, respectively

Irisin, through integrin αVβ5, concurrently maintains ER homeostasis and enhances AMPK activity, thereby alleviating emphysema and inflammatory responses triggered by the combination of CSE and LPS (Fig. 10). These results collectively identify an innovative therapeutic target for irisin in the treatment of AECOPD and provide a theoretical basis for further exploration of the clinical application potential of irisin in AECOPD intervention.

## Supplementary Information


Supplementary Material 1.


## Data Availability

All additional files are included in the manuscript. Further inquiries can be directed to the corresponding authors.

## References

[1] Agusti A, Melen E, DeMeo DL, Breyer-Kohansal R, Faner R. Pathogenesis of chronic obstructive pulmonary disease: understanding the contributions of gene-environment interactions across the lifespan. Lancet Respir Med. 2022;10(5):512–24. 35427533 10.1016/S2213-2600(21)00555-5 PMC11428195

[2] Celli B, Fabbri L, Criner G, Martinez FJ, Mannino D, Vogelmeier C, Montes de Oca M, Papi A, Sin DD, Han MK, et al. Definition and Nomenclature of Chronic Obstructive Pulmonary Disease: Time for Its Revision. Am J Respir Crit Care Med. 2022;206(11):1317–25. 35914087 10.1164/rccm.202204-0671PP PMC9746870

[3] Cao Z, He L, Luo Y, Tong X, Zhao J, Huang K, Chen Q, Jiao L, Liu Y, Geldsetzer P, et al. Burden of chronic respiratory diseases and their attributable risk factors in 204 countries and territories, 1990–2021: Results from the global burden of disease study 2021. Chin Med J Pulm Crit Care Med. 2025;3(2):100–10. 40677417 10.1016/j.pccm.2025.05.005 PMC12266273

[4] Zhang J, Xu N, Zheng D. Effect of High-quality nursing care on patients with acute exacerbation of chronic obstructive pulmonary disease complicated with respiratory failure: an observational cohort study. Appl Bionics Biomech. 2022; 2022:9440899. 35733447 10.1155/2022/9440899 PMC9208997

[5] Wu YK, Su WL, Yang MC, Chen SY, Wu CW, Lan CC. Characterization Associated with the Frequent Severe Exacerbator Phenotype in COPD Patients. Int J Chron Obstruct Pulmon Dis. 2021;16:2475–85. 34511892 10.2147/COPD.S317177 PMC8416186

[6] Bostrom P, Wu J, Jedrychowski MP, Korde A, Ye L, Lo JC, Rasbach KA, Bostrom EA, Choi JH, Long JZ, et al. A PGC1-alpha-dependent myokine that drives brown-fat-like development of white fat and thermogenesis. Nature. 2012;481(7382):463–8. 22237023 10.1038/nature10777 PMC3522098

[7] Kim H, Wrann CD, Jedrychowski M, Vidoni S, Kitase Y, Nagano K, Zhou C, Chou J, Parkman VA, Novick SJ, et al. Irisin Mediates Effects on Bone and Fat via alphaV Integrin Receptors. Cell. 2018;175(7):1756–68. e1717. 30550785 10.1016/j.cell.2018.10.025 PMC6298040

[8] Chen K, Xu Z, Liu Y, Wang Z, Li Y, Xu X, Chen C, Xia T, Liao Q, Yao Y, et al. Irisin protects mitochondria function during pulmonary ischemia/reperfusion injury. Sci Transl Med. 2017;9(418):eaao6298. 10.1126/scitranslmed.aao6298 PMC5969805 29187642

[9] Sugiyama Y, Asai K, Yamada K, Kureya Y, Ijiri N, Watanabe T, Kanazawa H, Hirata K. Decreased levels of irisin, a skeletal muscle cell-derived myokine, are related to emphysema associated with chronic obstructive pulmonary disease. Int J Chron Obstruct Pulmon Dis. 2017;12:765–72. 28424548 10.2147/COPD.S126233 PMC5344413

[10] Kubo H, Asai K, Kojima K, Sugitani A, Kyomoto Y, Okamoto A, Yamada K, Ijiri N, Watanabe T, Hirata K, et al. Exercise Ameliorates Emphysema Of Cigarette Smoke-Induced COPD In Mice Through The Exercise-Irisin-Nrf2 Axis. Int J Chron Obstruct Pulmon Dis. 2019;14:2507–16. 31814716 10.2147/COPD.S226623 PMC6862806

[11] Ma J, Han Z, Jiao R, Yuan G, Ma C, Yan X, Meng A. Irisin Ameliorates PM2.5-Induced Acute Lung Injury by Regulation of Autophagy Through AMPK/mTOR Pathway. J Inflamm Res. 2023;16:1045–57. 36936349 10.2147/JIR.S390497 PMC10018221

[12] Jiao R, Han Z, Ma J, Wu S, Wang Z, Zhou G, Liu X, Li J, Yan X, Meng A. Irisin attenuates fine particulate matter induced acute lung injury by regulating Nod2/NF-kappaB signaling pathway. Immunobiology. 2023;228(3):152358. 37003140 10.1016/j.imbio.2023.152358

[13] Han Z, Ma J, Han Y, Yuan G, Jiao R, Meng A. Irisin attenuates acute lung injury by suppressing the pyroptosis of alveolar macrophages. Int J Mol Med. 2023;51(4):32. 10.3892/ijmm.2023.5235 PMC10035983 36896789

[14] Chen Z, Wu S, Bai Z, Sun T, Ma C, Chen Y, Jiang P, Meng A. Irisin activates the AMPK-Beclin1 signaling pathway to regulate pulmonary autophagy induced by CSE + LPS. Sci Rep. 2025;15(1):44320. 41423545 10.1038/s41598-025-31824-2 PMC12722212

[15] Wales AM, Zhou TE, Draga-Coleta H, Gorgulla SV, Blackmore C, Mittenbuhler KA, Kim MJ, Bogoslavski CR, Zhang D. Irisin acts through its integrin receptor in a two-step process involving extracellular Hsp90alpha. Mol Cell. 2023;83(11):1903–20. e1912. 37267907 10.1016/j.molcel.2023.05.008 PMC10984146

[16] Bai Z, Chen Z, Sun T, Ma C, Meng A. Potential role of integrin alphaV as irisin receptor and advances in research. Peptides. 2025;193:171439. 40953608 10.1016/j.peptides.2025.171439

[17] Campbell ID, Humphries MJ. Integrin structure, activation, and interactions. Cold Spring Harb Perspect Biol. 2011;3(3):a004994. 10.1101/cshperspect.a004994 PMC3039929 21421922

[18] Ushioda R, Nagata K. Redox-Mediated Regulatory Mechanisms of Endoplasmic Reticulum Homeostasis. Cold Spring Harb Perspect Biol. 2019;11(5):a033910. 10.1101/cshperspect.a033910 PMC6496348 30396882

[19] Barreiro E, Salazar-Degracia A, Sancho-Munoz A, Aguilo R, Rodriguez-Fuster A, Gea J. Endoplasmic reticulum stress and unfolded protein response in diaphragm muscle dysfunction of patients with stable chronic obstructive pulmonary disease. J Appl Physiol (1985). 2019;126(6):1572–86. 30998124 10.1152/japplphysiol.00670.2018

[20] Wang HL, Chen FQ, Wu LJ. Ephedrine ameliorates chronic obstructive pulmonary disease (COPD) through restraining endoplasmic reticulum (ER) stress in vitro and in vivo. Int Immunopharmacol. 2022;103:107842. 34953449 10.1016/j.intimp.2021.107842

[21] Taylor CW, Tovey SC. IP(3) receptors: toward understanding their activation. Cold Spring Harb Perspect Biol. 2010;2(12):a004010. 20980441 10.1101/cshperspect.a004010 PMC2982166

[22] Coe H. M <>Michalak 2009 Calcium binding chaperones of the endoplasmic reticulum. Gen Physiol Biophys 28 Spec No Focus F96–103. 20093733

[23] Chung KF, Adcock IM. Multifaceted mechanisms in COPD: inflammation, immunity, and tissue repair and destruction. Eur Respir J. 2008;31(6):1334–56. 18515558 10.1183/09031936.00018908

[24] Piperi C, Adamopoulos C, Dalagiorgou G, Diamanti-Kandarakis E, Papavassiliou AG. Crosstalk between advanced glycation and endoplasmic reticulum stress: emerging therapeutic targeting for metabolic diseases. J Clin Endocrinol Metab. 2012;97(7):2231–42. 22508704 10.1210/jc.2011-3408

[25] Xie W, Xue Y, Song X, Zhang H, Chang G, Shen X. Forkhead box protein A2 alleviates toll-like receptor 4-mediated inflammation, endoplasmic reticulum stress, autophagy, and apoptosis induced by lipopolysaccharide in bovine hepatocytes. J Dairy Sci. 2023;106(3):2089–112. 36586798 10.3168/jds.2022-22252

[26] Naiel S, Tat V, Padwal M, Vierhout M, Mekhael O, Yousof T, Ayoub A, Abed S, Dvorkin-Gheva A, Ask K. Protein Misfolding and Endoplasmic Reticulum Stress in Chronic Lung Disease: Will Cell-Specific Targeting Be the Key to the Cure? Chest. 2020;157(5):1207–20. 31778676 10.1016/j.chest.2019.11.009

[27] Hetz C, Zhang K, Kaufman RJ. Mechanisms, regulation and functions of the unfolded protein response. Nat Rev Mol Cell Biol. 2020;21(8):421–38. 32457508 10.1038/s41580-020-0250-z PMC8867924

[28] Yaguchi T, Chamoto K, Honjo T. Age-related immune states and PD-1 blockade: mechanisms and strategies for the elderly. J Immunother Cancer. 2025;13(12):e013783. 10.1136/jitc-2025-013783 PMC12673548 41330616

[29] Lin L, Hou G, Han D, Kang J, Wang Q. Ursolic Acid Protected Lung of Rats From Damage Induced by Cigarette Smoke Extract. Front Pharmacol. 2019;10:700. 31281258 10.3389/fphar.2019.00700 PMC6595172

[30] Lin L, Yin Y, Hou G, Han D, Kang J, Wang Q. Ursolic acid attenuates cigarette smoke-induced emphysema in rats by regulating PERK and Nrf2 pathways. Pulm Pharmacol Ther. 2017;44:111–21. 28347799 10.1016/j.pupt.2017.03.014

[31] Global strategy for prevention. diagnosis and management of COPD. https://goldcopd.org/2024-gold-report/.

[32] Guo B, Shi X, Jiang Q, Pan Y, Yang Y, Liu Y, Chen S, Zhu W, Ren L, Liang R, et al. Targeting Immunoproteasome in Polarized Macrophages Ameliorates Experimental Emphysema Via Activating NRF1/2-P62 Axis and Suppressing IRF4 Transcription. Adv Sci (Weinh). 2024;11(44):e2405318. 39356034 10.1002/advs.202405318 PMC11600198

[33] Kobayashi S, Fujinawa R, Ota F, Kobayashi S, Angata T, Ueno M, Maeno T, Kitazume S, Yoshida K, Ishii T, et al. A single dose of lipopolysaccharide into mice with emphysema mimics human chronic obstructive pulmonary disease exacerbation as assessed by micro-computed tomography. Am J Respir Cell Mol Biol. 2013;49(6):971–7. 23822858 10.1165/rcmb.2013-0074OC

[34] Al Hroob AM, Abukhalil MH, Hussein OE, Mahmoud AM. Pathophysiological mechanisms of diabetic cardiomyopathy and the therapeutic potential of epigallocatechin-3-gallate. Biomed Pharmacother. 2019;109:2155–72. 30551473 10.1016/j.biopha.2018.11.086

[35] Lu C, Fang S, Weng Q, Lv X, Meng M, Zhu J, Zheng L, Hu Y, Gao Y, Wu X, et al. Integrated analysis reveals critical glycolytic regulators in hepatocellular carcinoma. Cell Commun Signal. 2020;18(1):97. 32576292 10.1186/s12964-020-00539-4 PMC7310503

[36] Song Y, Han S, Liu L, Zheng L, Wang J, Cui Z, Yan X. circ_0000554 promotes macrophage M2 polarization mediated by glycolytic reprogramming and aggravates COPD injury. Toxicol Lett. 2025;410:147–58. 40555339 10.1016/j.toxlet.2025.06.014

[37] Wang Y, Liao S, Pan Z, Jiang S, Fan J, Yu S, Xue L, Yang J, Ma S, Liu T, et al. Hydrogen sulfide alleviates particulate matter-induced emphysema and airway inflammation by suppressing ferroptosis. Free Radic Biol Med. 2022;186:1–16. 35490984 10.1016/j.freeradbiomed.2022.04.014

[38] Qi C, Zhou J, Wang Z, Fang X, Li D, Jin Y, Song J. Cigarette smoke extract combined with lipopolysaccharide reduces OCTN1/2 expression in human alveolar epithelial cells in vitro and rat lung in vivo under inflammatory conditions. Int Immunopharmacol. 2020;87:106812. 32707498 10.1016/j.intimp.2020.106812

[39] Diler E, Schwarz M, Nickels R, Menger MD, Beisswenger C, Meier C, Tschernig T. Influence of external calcium and thapsigargin on the uptake of polystyrene beads by the macrophage-like cell lines U937 and MH-S. BMC Pharmacol Toxicol. 2014;15:16. 24646666 10.1186/2050-6511-15-16 PMC3994969

[40] Kovuru N, Mochizuki-Kashio M, Menna T, Jeffrey G, Hong Y, Me Yoon Y, Zhang Z, Kurre P. Deregulated protein homeostasis constrains fetal hematopoietic stem cell pool expansion in Fanconi anemia. Nat Commun. 2024;15(1):1852. 38424108 10.1038/s41467-024-46159-1 PMC10904799

[41] Chung J, Serezani CH, Huang SK, Stern JN, Keskin DB, Jagirdar R, Brock TG, Aronoff DM, Peters-Golden M. Rap1 activation is required for Fc gamma receptor-dependent phagocytosis. J Immunol. 2008;181(8):5501–9. 18832707 10.4049/jimmunol.181.8.5501 PMC3077557

[42] Li Z, Jiang Q, Wei J, Dang D, Meng Z, Wu H. Piezo1 promotes the progression of necrotizing enterocolitis by activating the Ca2(+)/CaMKII-dependent pathway. Commun Biol. 2025;8(1):417. 40074811 10.1038/s42003-025-07821-6 PMC11904196

[43] Liang L, He C, Han X, Liu J, Yang L, Chang F, Zhang Y, Lin J. Zuojin Pill Alleviates Precancerous Lesions of Gastric Cancer by Modulating the MEK/ERK/c-Myc Pathway: An Integrated Approach of Network Pharmacology, Molecular Dynamics Simulation, and Experimental Validation. Drug Des Devel Ther. 2024;18:5905–29. 39679136 10.2147/DDDT.S487371 PMC11646374

[44] Lill MA, Danielson ML. Computer-aided drug design platform using PyMOL. J Comput Aided Mol Des. 2011;25(1):13–9. 21053052 10.1007/s10822-010-9395-8

[45] Van Der Spoel D, Lindahl E, Hess B, Groenhof G, Mark AE, Berendsen HJ. GROMACS: fast, flexible, and free. J Comput Chem. 2005;26(16):1701–18. 16211538 10.1002/jcc.20291

[46] Lindorff-Larsen K, Piana S, Palmo K, Maragakis P, Klepeis JL, Dror RO, Shaw DE. Improved side-chain torsion potentials for the Amber ff99SB protein force field. Proteins. 2010;78(8):1950–8. 20408171 10.1002/prot.22711 PMC2970904

[47] Genheden S, Ryde U. The MM/PBSA and MM/GBSA methods to estimate ligand-binding affinities. Expert Opin Drug Discov. 2015;10(5):449–61. 25835573 10.1517/17460441.2015.1032936 PMC4487606

[48] Lee AS. The glucose-regulated proteins: stress induction and clinical applications. Trends Biochem Sci. 2001;26(8):504–10. 11504627 10.1016/s0968-0004(01)01908-9

[49] Thastrup O, Cullen PJ, Drobak BK, Hanley MR, Dawson AP. Thapsigargin, a tumor promoter, discharges intracellular Ca2 + stores by specific inhibition of the endoplasmic reticulum Ca2(+)-ATPase. Proc Natl Acad Sci U S A. 1990;87(7):2466–70. 2138778 10.1073/pnas.87.7.2466 PMC53710

[50] Collatz MB, Rudel R, Brinkmeier H. Intracellular calcium chelator BAPTA protects cells against toxic calcium overload but also alters physiological calcium responses. Cell Calcium. 1997;21(6):453–9. 9223681 10.1016/s0143-4160(97)90056-7

[51] Cuttitta G, Ferraro M, Cibella F, Alfano P, Bucchieri S, Patti AM, Muratori R, Pace E, Bruno A. Relationship among Body Composition, Adipocytokines, and Irisin on Exercise Capacity and Quality of Life in COPD: A Pilot Study. Biomolecules. 2022;13(1):48. 10.3390/biom13010048 PMC9855916 36671433

[52] Papp C, Pak K, Erdei T, Juhasz B, Seres I, Szentpeteri A, Kardos L, Szilasi M, Gesztelyi R, Zsuga J. Alteration of the irisin-brain-derived neurotrophic factor axis contributes to disturbance of mood in COPD patients. Int J Chron Obstruct Pulmon Dis. 2017;12:2023–33. 28744117 10.2147/COPD.S135701 PMC5511021

[53] Checa M, Hagood JS, Velazquez-Cruz R, Ruiz V, Garcia-De-Alba C, Rangel-Escareno C, Urrea F, Becerril C, Montano M, Garcia-Trejo S, et al. Cigarette Smoke Enhances the Expression of Profibrotic Molecules in Alveolar Epithelial Cells. PLoS ONE. 2016;11(3):e0150383. 26934369 10.1371/journal.pone.0150383 PMC4775036

[54] Tatler AL, John AE, Jolly L, Habgood A, Porte J, Brightling C, Knox AJ, Pang L, Sheppard D, Huang X, et al. Integrin alphavbeta5-mediated TGF-beta activation by airway smooth muscle cells in asthma. J Immunol. 2011;187(11):6094–107. 22025551 10.4049/jimmunol.1003507 PMC3242066

[55] Wang Y, Tian M, Tan J, Pei X, Lu C, Xin Y, Deng S, Zhao F, Gao Y, Gong Y. Irisin ameliorates neuroinflammation and neuronal apoptosis through integrin alphaVbeta5/AMPK signaling pathway after intracerebral hemorrhage in mice. J Neuroinflammation. 2022;19(1):82. 35392928 10.1186/s12974-022-02438-6 PMC8988353

[56] Zhang Q, Xiang S, Chen X, Rong Y, Huang L, Chen Z, Yao K, Chen W, Deng C, Wang J. Irisin attenuates acute glaucoma-induced neuroinflammation by activating microglia-integrin alphaVbeta5/AMPK and promoting autophagy. Int Immunopharmacol. 2024;138:112545. 38955026 10.1016/j.intimp.2024.112545

[57] Zhang H, Zhu DS, Zhu J. Family-wide analysis of integrin structures predicted by AlphaFold2. Comput Struct Biotechnol J. 2023;21:4497–507. 37753178 10.1016/j.csbj.2023.09.022 PMC10518446

[58] Lu CH, Lee CE, Zhang W, Yang Y, Valencia LA, You H, Tsai CT, Cui B. FCHo2, not talin, enables inside-out activation of integrin avbeta5 in curved adhesions. Nat Commun. 2026;17:2419. 41651837 10.1038/s41467-026-68942-y PMC12988159

[59] Kinashi T. Intracellular signalling controlling integrin activation in lymphocytes. Nat Rev Immunol. 2005;5(7):546–59. 15965491 10.1038/nri1646

[60] Li RL, Zhuo CL, Yan X, Li H, Lin L, Li LY, Jiang Q, Zhang D, Wang XM, Liu LL, et al. Irisin attenuates vascular remodeling in hypertensive mice induced by Ang II by suppressing Ca(2+)-dependent endoplasmic reticulum stress in VSMCs. Int J Biol Sci. 2024;20(2):680–700. 38169582 10.7150/ijbs.84153 PMC10758105

[61] Preissler S, Ron D. Early Events in the Endoplasmic Reticulum Unfolded Protein Response. Cold Spring Harb Perspect Biol. 2019;11(4):a033894. 30396883 10.1101/cshperspect.a033894 PMC6442202

[62] Peng C, Wang J, Wang S, Zhao Y, Wang H, Wang Y, Ma Y, Yang J. Endoplasmic Reticulum Stress: Triggers Microenvironmental Regulation and Drives Tumor Evolution. Cancer Med. 2025;14(5):e70684. 40035165 10.1002/cam4.70684 PMC11877002

[63] Bi J, Zhang J, Ren Y, Du Z, Li T, Wang T, Zhang L, Wang M, Wu Z, Lv Y, et al. Irisin reverses intestinal epithelial barrier dysfunction during intestinal injury via binding to the integrin alphaVbeta5 receptor. J Cell Mol Med. 2020;24(1):996–1009. 31701659 10.1111/jcmm.14811 PMC6933384

[64] Bi J, Zhang J, Ren Y, Du Z, Zhang Y, Liu C, Wang Y, Zhang L, Shi Z, Wu Z, et al. Exercise hormone irisin mitigates endothelial barrier dysfunction and microvascular leakage-related diseases. JCI Insight. 2020;5(13):e136277. 10.1172/jci.insight.136277 PMC7406290 32516137

